# m^6^A-induced lncDBET promotes the malignant progression of bladder cancer through FABP5-mediated lipid metabolism

**DOI:** 10.7150/thno.71456

**Published:** 2022-08-29

**Authors:** Peihua Liu, Benyi Fan, Belaydi Othmane, Jiao Hu, Huihuang Li, Yu Cui, Zhenyu Ou, Jinbo Chen, Xiongbing Zu

**Affiliations:** 1 Department of Urology, Xiangya Hospital, Central South University, Changsha, Hunan 410008, PR China.; 2 National Clinical Research Center for Geriatric Disorders, Xiangya Hospital, Central South University, Changsha, Hunan 410008, PR China.

**Keywords:** Bladder cancer, m^6^A, METTL14, lncDBET, FABP5

## Abstract

The limited effect of adjuvant therapy for advanced bladder cancer (BCa) leads to a poor prognosis. Increasing evidence has shown that RNA N6-methyladenosine (m^6^A) modification plays important functional roles in tumorigenesis. Nevertheless, the role and mechanism of m6A-modified noncoding RNAs (ncRNAs) in BCa remain largely unknown.

**Methods:** RT-PCR, western blotting and ONCOMINE dataset were used to determine the dominant m^6^A-related enzyme in BCa. M^6^A-lncRNA epitranscriptomic microarray was used to screen candidate targets of METTL14. RT-PCR, MeRIP and TCGA dataset were carried out to confirm the downstream target of METTL14. CHIRP/MS was conducted to identify the candidate proteins binding to lncDBET. RT-PCR, western blotting, RIP and KEGG analysis were used to confirm the target of lncDBET. The levels of METTL14, lncDBET and FABP5 were tested *in vitro* and *in vivo*. CCK-8, EdU, transwell and flow cytometry assays were performed to determine the oncogenic function of METTL14, lncDBET and FABP5, and their regulatory networks.

**Results**: We identified that the m^6^A level of total RNA was elevated and that METTL14 was the dominant m6A-related enzyme in BCa. m^6^A modification mediated by METTL14 promoted the malignant progression of BCa by promoting the expression of lncDBET. Upregulated lncDBET activated the PPAR signalling pathway to promote the lipid metabolism of cancer cells through direct interaction with FABP5, thus promoting the malignant progression of BCa *in vitro* and *in vivo*.

**Conclusions**: Our study establishes METTL14/lncDBET/FABP5 as a critical oncogenic axis in BCa.

## Introduction

It is estimated that in 2022, bladder cancer (BCa) will rank fourth and eighth in terms of new cases and deaths among all-male malignant tumours in the US 1. BCa exhibits rapid progression and a high recurrence rate; 25%-30% of BCa cases are considered muscle-invasive bladder cancer (MIBC) at the time of diagnosis, and 5% of cases show distant metastasis 2. Even though some patients with advanced BCa can benefit from the diversification of neoadjuvant chemotherapy, immunotherapy or Bacillus Calmette-Guerin (BCG) therapy, the overall prognosis is still poor. Therefore, exploring new therapeutic targets for BCa is of great significance.

Among all the RNA modification modes, N6-methyladenosine (m^6^A) is the most abundant posttranscriptional modification detected in eukaryotic mRNAs and noncoding RNAs (ncRNAs) 3, 4. m^6^A modification is dynamically reversible, and disruption of the modification balance triggers the occurrence and progression of human cancers 4. Many studies have reported on the regulation of BCa progression by m^6^A-modified mRNAs. For example, METTL3-mediated m^6^A modification promotes the progression of BCa by activating the AFF4/NF-κB/MYC signalling pathway 5, and glycolysis mediated by ALKBH5-CK2α can sensitize BCa cells to chemotherapy 6. Nevertheless, the role and mechanism of m^6^A-modified ncRNAs in BCa remain largely unknown and need to be clarified.

Long noncoding RNAs (lncRNAs), a class of transcripts more than 200 nt in length, have limited or no protein-coding potential 7. LncRNAs have been widely shown to play both oncogenic and antioncogenic roles in human cancers, and the mechanisms by which they regulate gene expression include chromatin modification, protein binding, cytoplasmic scaffolding and RNA decay 8-11. However, additional regulatory mechanisms of lncRNAs in human cancers may be discovered in the future with the advancement of biotechnology. To date, more than 100 lncRNAs, including MALAT1, UCA1, H19, NORAD and TUG1, have been found to be involved in BCa 12-14. Therefore, additional functions and mechanisms of lncRNAs in BCa should be explored.

As an essential component of cell metabolism, fatty acids (FAs) are involved in cellular signalling pathways and act to maintain physiological functions. However, metabolic dysregulation has been confirmed to be an important hallmark of cancers 15. FAs are required for cancer cells to supply signaling molecules, cellular structural elements and sources of metabolic energy 16. Altered lipid metabolism in BCa has been detected, and the FA composition in BCa differs from that in normal urothelial cells 17. Fatty acid binding proteins (FABPs) are a family of small, highly conserved, cytoplasmic proteins that can bind long-chain FAs 18. FABPs play crucial roles in FA uptake, transport, and metabolism, and thus reflect the lipid metabolism level of cells 19, 20.

In this study, we found that m^6^A modification mediated by METTL14 promotes the malignant progression of BCa by promoting the expression of lncDBET. LncDBET directly interacts with FABP5 and activates the PPAR signalling pathway to promote the malignant progression of BCa. Overall, we highlight the role of m^6^A-modified lncDBET in BCa and identify a promising clinical marker of BCa.

## Results

### METTL14 is overexpressed in BCa and promotes the malignant progression of BCa

**Figure 1:**
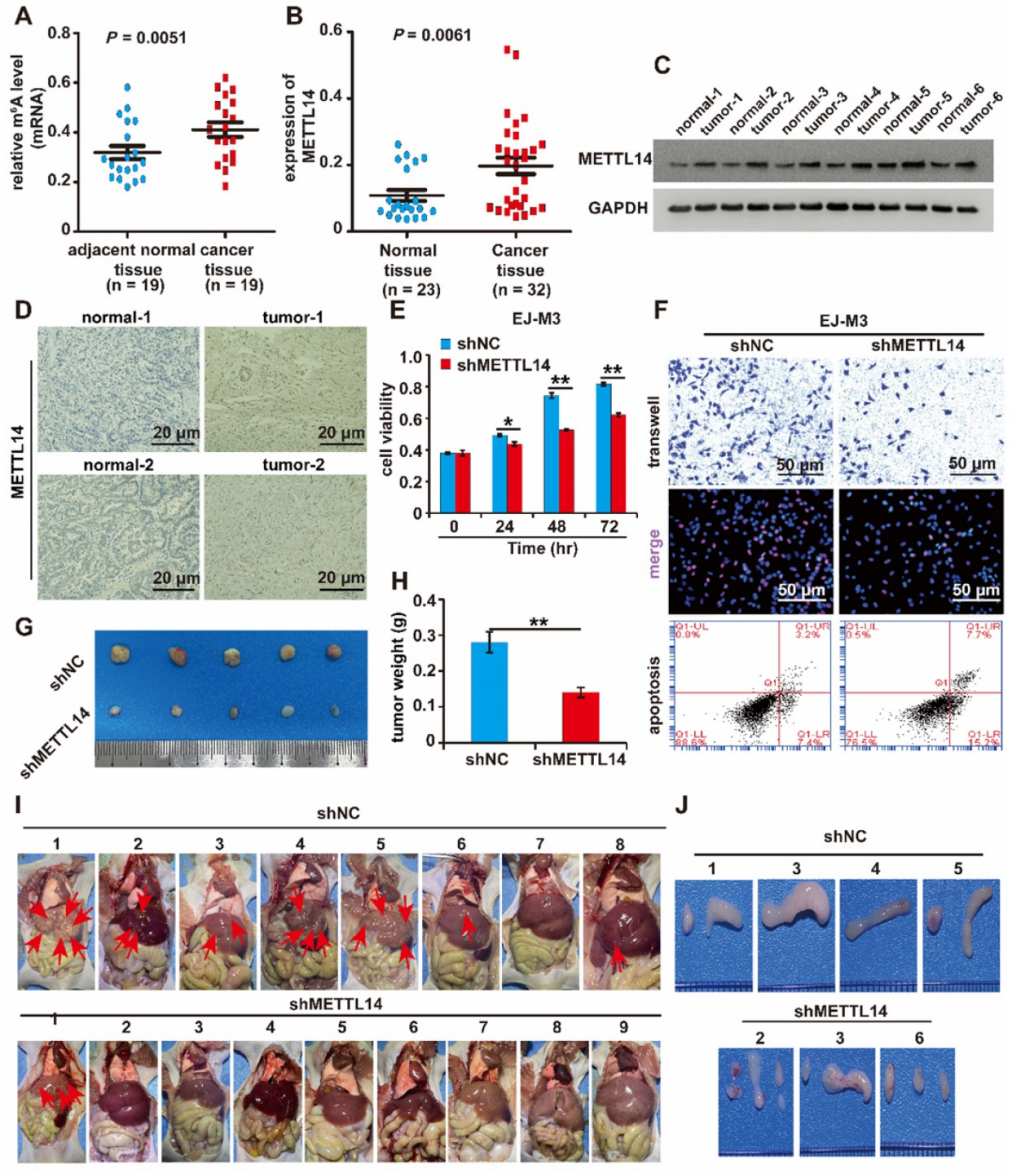
** Identification of METTL14 as the prominent m^6^A-associated enzyme in BCa.** (A) Relative m6A level in BCa tissues (n = 19) and adjacent normal tissues (n = 19). (B) qPCR analysis of the(D) Immunohistochemistry analysis of METTL14 in two paired cancer and adjacent normal tissues of BCa. The bar was 20 μm. (E) CCK8 assay for the viability of EJ-M3 cell lines respectively transfected with shMETTL14 and pHIV-METTL14 and control shRNA(shNC)/pHIV-RNA (pHIV-NC). (F) Transwell, EdU and Flow cytometry assays for the migration, proliferation and apoptosis of EJ-M3 cell lines respectively transfected with shMETTL14 and pHIV-METTL14 and control shRNA (shNC)/pHIV-RNA (pHIV-NC). UL, fragment and damaged cells; UR, late apoptosis and dead cells; LL, normal cells of negative control group; LR, early apoptotic cells. (G) Representative images of the xenograft tumors in Balbc nu/nu nude mice subcutaneously injected EJ-M3 cells transfected with shMETTL14 or shNC. (H) The relative weight of tumors in Balbc nu/nu nude mice subcutaneously injected EJ-M3 cells transfected with shMETTL14 or shNC. (I) Representative images of the metastasis tumors in Balbc nu/nu nude mice injected EJ-M3 cells transfected with shMETTL14 or shNC through caudal vein. (J) Images of the lymph glands in metastasis tumor mice model injected EJ-M3 cells transfected with shMETTL14 or shNC through caudal vein.

To determine the role of RNA m6A modification in BCa, the level of m^6^A modification in BCa and normal samples was measured. The results revealed that the level of m^6^A modification was increased in BCa samples compared to normal samples (Figure 1A). Additionally, we measured the main m^6^A-associated methyltransferases (METTL3, METTL14 and WTAP) and demethylases (FTO and ALKBH5) in BCa and normal samples. The results of the qPCR analysis revealed that the level of METTL14 mRNA transcripts was significantly elevated (Figure 1B). However, no alteration was observed in the expression of METTL3, FTO and ALKBH5 in BCa samples compared to normal samples (Figure S1A-D). Next, the western blotting results showed that the quantity of METTL14 protein was significantly increased in BCa samples compared to normal samples (Figure 1C). Furthermore, the expression level of METTL14 was validated in BCa and adjacent normal tissues by immunohistochemistry. The results demonstrated that METTL14 expression was significantly increased in BCa tissues (Figure 1D).

In addition, we analysed METTL14 expression in the bladder based on the data of the *LEE Bladder* in the ONCOMINE database (Reporter: ILMN_1673037), and the result was consistent with that of our research, which showed that METTL14 expression was increased in BCa samples compared to normal bladder samples (Figure S1E). Furthermore, we measured the expression of METTL14 in the normal urothelial cell line SVHUC-1 and five BCa cell lines (UMUC3, 5637, T24, J82 and EJ-M3). The results of qPCR and western blotting assays showed that both the mRNA and protein levels of METTL14 were elevated in BCa cell lines compared to the normal urothelial cell line SVHUC-1 (Figure S1F-G). Collectively, these results revealed that RNA m^6^A modification was involved and that METTL14 was overexpressed in BCa.

Based on what we noted above, METTL14 was upregulated in BCa samples and cell lines. We speculated that METTL14 serves as a tumour promoter in BCa. To determine the function of METTL14 in BCa, METTL14 overexpression and silencing systems were constructed in J82 and EJ-M3 cells (Figure S1H-K). The function of METTL14 in cell viability, proliferation, migration and apoptosis was assessed. As expected, the ectopic expression of METTL14 markedly enhanced the viability, proliferation and migration ability of J82 cells, while the depletion of METTL14 significantly decreased the viability, proliferation, and migration ability of EJ-M3 cells and increased the apoptosis ability of EJ-M3 cells (Figure 1E-F, Figure S1L-M).

Furthermore, we constructed a METTL14 deletion EJ-M3 cell model. The mouse models of subcutaneous tumours were constructed by subcutaneous inoculation of the cells. Consistent with the above in vitro results, silencing METTL14 resulted in significant decreases in tumour volume and weight (Figure 1G-H).

Moreover, tail vein injection models were also constructed by tail vein injection of the cell model. The results demonstrated that of the 10 mice in the control group, 2 died, and 7 out of the 8 remaining mice had liver metastases (Figure 1I). while among the 10 METTL14 deletion mice, 1 died, and only 1 out of the 9 remaining mice had liver metastases (Figure 1I). The HE staining results of the liver tissue also showed that METTL14 deletion significantly reduced the liver metastasis rate (Figure S1N). In the control group, 4 had lymphatic metastasis, while in the shMETTL14 group, 3 had lymphatic metastasis (Figure 1J). Collectively, these results demonstrated that METTL14 depletion inhibited the liver and lymphatic metastasis rates of BCa.

Overall, these results showed that METTL14 promotes the malignant progression of BCa *in vitro* and* in vivo*.

### Analysis of downstream targets of METTL14 in BCa

**Figure 2:**
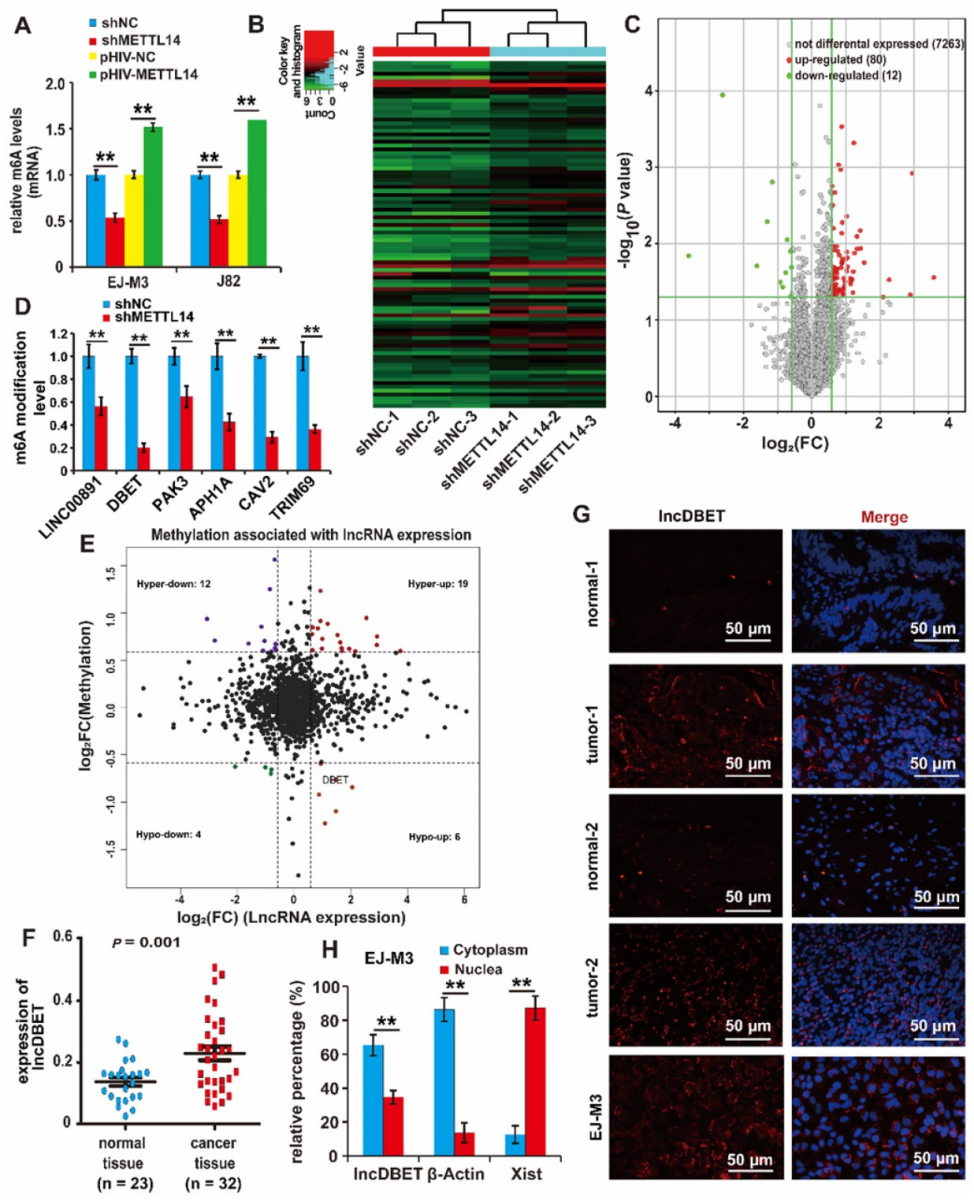
** Identification of downstream target lncRNAs of METTL14 in BCa.** (A) Relative m6A levels in EJ-M3 and J82 cell lines transfected with shMETTL14 and pHIV-METTL14, and control shRNA (shNC)/pHIV-RNA (pHIV-NC)(B) Hierarchical clustering of m6A-lncRNA epitranscriptomic microarray of three groups of matched shNC and shMETTL14 EJ-M3 cell lines. (C) Volcano plots for differential expressed m6A modification transcripts (lncRNAs) between shNC and shMETTL14 EJ-M3 cells. (|Fold Change| ≥ 1.5, P < 0.05). (D) qPCR analysis of the m6A modification level of 6 candidate lncRNAs. (E) Volcano plots for comprehensive analysis of BCa m6A-lncRNA & lncRNA expression microarray (|FC|≥1.5). Hyper, lncRNAs of up-regulated m6A modification; Hypo, lncRNAs of down-regulated m6A modification; up, up-regulated lncRNAs in terms of quantity; down, down-regulated lncRNAs in terms of quantity. (F) qPCR analysis of lncDBET in BCa tissues and normal bladder tissues. (G) Localization analysis of lncDBET by fluorescence in situ hybridization (FISH) in BCa tissues, normal bladder tissues, and EJ-M3 cells. Cell nuclei were counterstained with Hoechst (blue). Each experiment was repeated a minimum of three times. The symbol * denotes a significant difference (P < 0.05), while ** represents a highly significant difference (P < 0.01). (H) Localization analysis of lncDBET by biochemical fractionation in EJ-M3 cells. β-Actin (mostly in the cytoplasm) and Xist (mostly in the nuclea) are as control teams.

To identify whether METTL14 regulates RNA m^6^A modification in BCa cell lines, total RNA m^6^A modification levels were assessed; the results showed that ectopic expression of METTL14 increased the modification level. Conversely, the deletion of METTL14 decreased its modification level (Figure 2A).

To further investigate the regulatory role of METTL14 in BCa, three groups of matched shNC and shMETTL14 BCa EJ-M3 cell lines were prepared, and an m6A-lncRNA epitranscriptomic microarray was used to screen candidate target lncRNAs of METTL14 (Figure 2B). Overall, the results identified 80 upregulated m^6^A modification transcripts and 12 downregulated transcripts (Figure 2C) (|FC| ≥ 1.5, *P* < 0.05). Of the 12 downregulated lncRNAs (Table S1), only 6 lncRNAs (APH1A, LINC00891, DBET, TRIM69, CAV2 and PAK3) have been studied before and proven to be functional. To test the reliability of the epitranscriptomic microarray results, a qPCR assay was employed to measure the m^6^A modification level of these 6 lncRNAs. The results demonstrated that silencing METTL14 decreased the m^6^A modification level of all these lncRNAs, especially lncDBET (Figure 2D).

Moreover, a comprehensive analysis of the BCa **m6A-lncRNA microarray** combined with the **lncRNA expression microarray** used previously 21 was made (|FC| ≥ 1.5), and there were 6 lncRNAs in the Hypo-up quadrant (Figure 2E, Table S2). This group of 6 lncRNAs was compared with the group of 6 downregulated m^6^A modification lncRNAs that have been studied before and proved to be functional, and only lncDBET was repeated. Analysis from The Cancer Genome Atlas (TCGA) database also indicated that only lncDBET was overexpressed in BCa tissues (Table S3). Furthermore, lncDBET was confirmed to be overexpressed in BCa tissues and cell lines (Figure 2F, Figure S2A).

Moreover, the subcellular localization of lncDBET was tested by biochemical fractionation and fluorescence in situ hybridization (FISH) assays in BCa tissues and cell lines. The results demonstrated that lncDBET was localized in both the cytoplasm and nucleus, especially in the cytoplasm (Figure 2G-H, Figure S2B-C).

### METTL14 affects the stability of lncDBET transcripts by modulating their m^6^A modification

**Figure 3:**
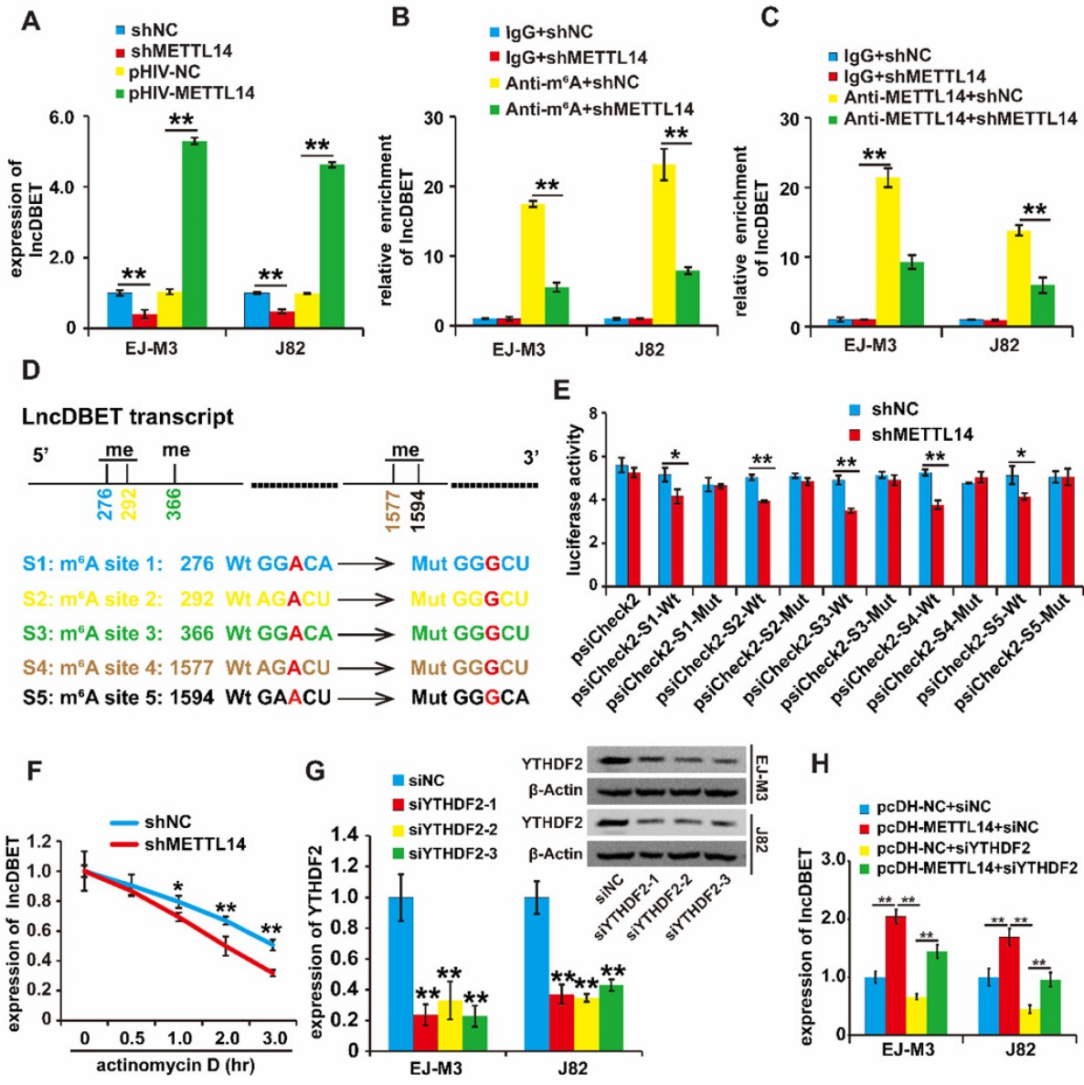
** Regulation analysis of lncDBET by METTL14 in m^6^A modification.** (A) qPCR analysis of expression level of lncDBET regulated by METTL14 in EJ-M3 and J82 cell lines. (B) MeRIP-qPCR analysis of lncDBET regulated by METTL14 in EJ-M3 and J82 cell lines. (C) METTL14-RIP-qPCR analysis of lncDBET regulated by METTL14 in EJ-M3 and J82 cell lines. (D) The predicted m6A sites in lncDBET transcript and the mutated m6A sites of lncDBET. (E) The inhibitory role of shMETTL14 in the luciferase activity inserted the lncDBET with mutant type (Mut) m6A sites in EJ-M3. (F) RNA stability analysis of lncDBET regulated by METTL14 in EJ-M3. (G) qPCR and Western blotting assay analysis of the inhibitory roles of siYTHDF2 on YTHDF2 expression. (H) qPCR assay was used to measure the lncDBET expression in EJ-M3 and J82 cell lines. Each experiment was repeated a minimum of three times. The symbol * denotes a significant difference (P < 0.05), while ** represents a highly significant difference (P < 0.01).

To confirm the regulation of lncDBET by METTL14, we assessed the quantity of lncDBET transcripts in METTL14 overexpression and deletion cell lines, and the results demonstrated that ectopic expression of METTL14 increased the quantity of lncDBET transcripts. Conversely, silencing METTL14 lowered the quantity of lncDBET transcripts in EJ-M3 and J82 cells (Figure 3A), suggesting the positive regulation of lncDBET by METTL14.

Then, we investigated the involvement of m^6^A modification in the regulation of lncDBET by METTL14. The results of the MeRIP-qPCR assay revealed that the silencing of METTL14 reduced the enrichment of lncDBET (Figure 3B). Furthermore, the METTL14 RIP-qPCR assay results showed that the inhibition of METTL14 attenuated the enrichment of lncDBET (Figure 3C). Collectively, these results suggest that METTL14 directly interacts with lncDBET and inhibits lncDBET expression by reducing its m^6^A modification level.

To identify the m^6^A sites of lncDBET transcripts, we predicted m^6^A sites by using the following website: **http://www.cuilab.cn**. The results showed five possible m^6^A sites in the lncDBET transcripts (Table S4). To assess the role of m^6^A sites in the regulation of lncDBET transcripts, we mutated the m^6^A sites (Figure 3D) and employed a luciferase reporter assay (Figure 3E). The result revealed that silencing of METTL14 reduced the luciferase reporter activity of the reporter containing the lncDBET transcripts with wild type (Wt) m^6^A sites; moreover, there was no alteration in the luciferase reporter activity of the reporter containing the lncDBET transcripts with mutant type (Mut) m^6^A sites (Figure 3E). In addition, the RNA stability assay showed reduced stability of the lncDBET transcripts in cells with METTL14 knockdown (Figure 3F).

Systematic studies have illustrated the concept that the m6A “writer” and “reader” axes are likely to exist in human cancers 22, 23. To further dissect the mechanism underlying the regulation of lncDBET by METTL14-mediated m^6^A modification, we aimed to determine which “reader” was involved. Considering the main role of the “reader” YTHDF2 in RNA instability and its high expression level in BCa 22, 24, we first designed siRNAs to specifically target this reader (siYTHDF2s), and qPCR and western blotting assays demonstrated that all three siYTHDF2s efficiently inhibited the expression of YTHDF2 (Figure 3G). Furthermore, we divided the cells into four groups, and the qPCR assay results showed that silencing YTHDF2 inhibited lncDBET expression while simultaneously alleviating the METTL14-induced promotion of lncDBET expression (Figure 3H).

Overall, our study revealed that METTL14 improved the stability of lncDBET transcripts by modulating m^6^A modification to promote their expression.

### The role of the METTL14/lncDBET pathway in the malignant progression of BCa

**Figure 4:**
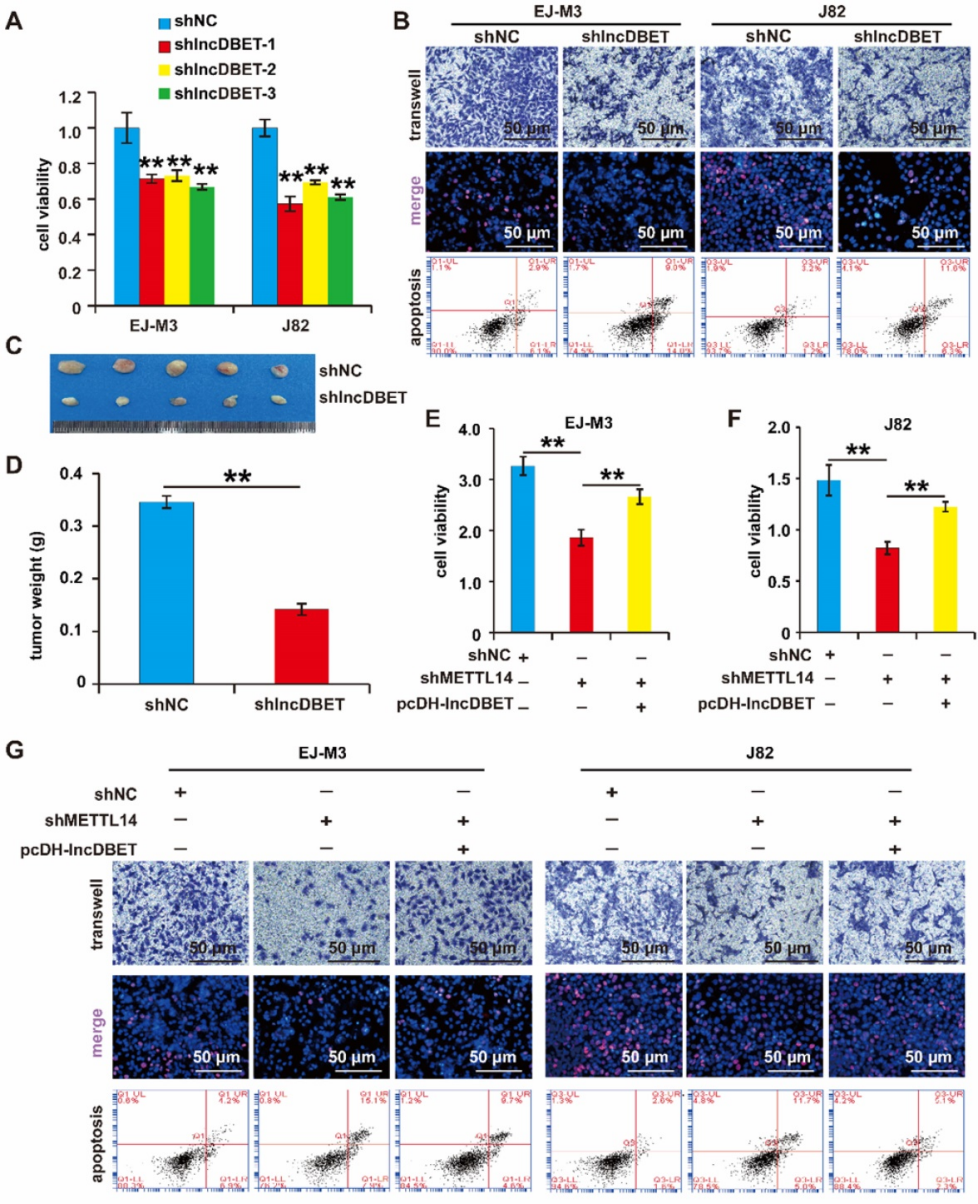
** Functional analysis of the METTL14/lncDBET pathway in progression of BCa.** (A) CCK8 assay for the viability of EJ-M3 and J82 cell lines transfected with shlncDBET. (B) Transwell, EdU and Flow cytometry assays for the migration, proliferation and apoptosis of EJ-M3 and J82 cell lines transfected with shlncDBET and control shRNA (shNC). (C) Representative images of the xenograft tumors in Balbc nu/nu nude mice subcutaneously injected EJ-M3 cells transfected with shlncDBET or shNC. (D) The relative weight of tumors in Balbc nu/nu nude mice subcutaneously injected EJ-M3 cells transfected with shlncDBET or shNC. (E-F) CCK8 assay for the viability of EJ-M3 and J82 cell lines transfected with shNC, shMETTL14 and shMETTL14+pcDH-lncDBET. (G) Transwell, EdU and Flow cytometry assays for the migration, proliferation and apoptosis of EJ-M3 and J82 cell lines transfected with shNC, shMETTL14 and shMETTL14+pcDH-lncDBET. Each experiment was repeated a minimum of three times. The symbol * denotes a significant difference (P < 0.05), while ** represents a highly significant difference (P < 0.01).

As mentioned above, lncDBET was overexpressed in BCa samples and cell lines. We speculated that lncDBET serves as a tumour promoter in BCa. To determine the function of lncDBET in BCa, we constructed a lncDBET overexpression and silencing system (Figure S2D-E). Then, the effects of lncDBET expression changes on cell viability, proliferation, migration and apoptosis were measured. As expected, the depletion of lncDBET significantly inhibited cell viability, proliferation and migration and promoted cell apoptosis (Figure 4A-B). The silencing of lncDBET resulted in significant decreases in tumour volume and weight (Figure 4C-D). Therefore, these results showed that lncDBET promotes the progression of BCa.

Of note, the above results revealed that both METTL14 and lncDBET promoted the survival, proliferation and migration and attenuated the apoptosis of BCa cells. In addition to this finding showing that lncDBET is regulated by METTL14, we demonstrated that the ectopic expression of lncDBET attenuates the repression of METTL14-induced inhibition of cell viability, proliferation and migration and promotion of cell apoptosis (Figure 4E-G).

Overall, METTL14 was shown to exert its oncogenic function by promoting the expression of lncDBET in BCa.

### Analysis of downstream targets of lncDBET in BCa

**Figure 5:**
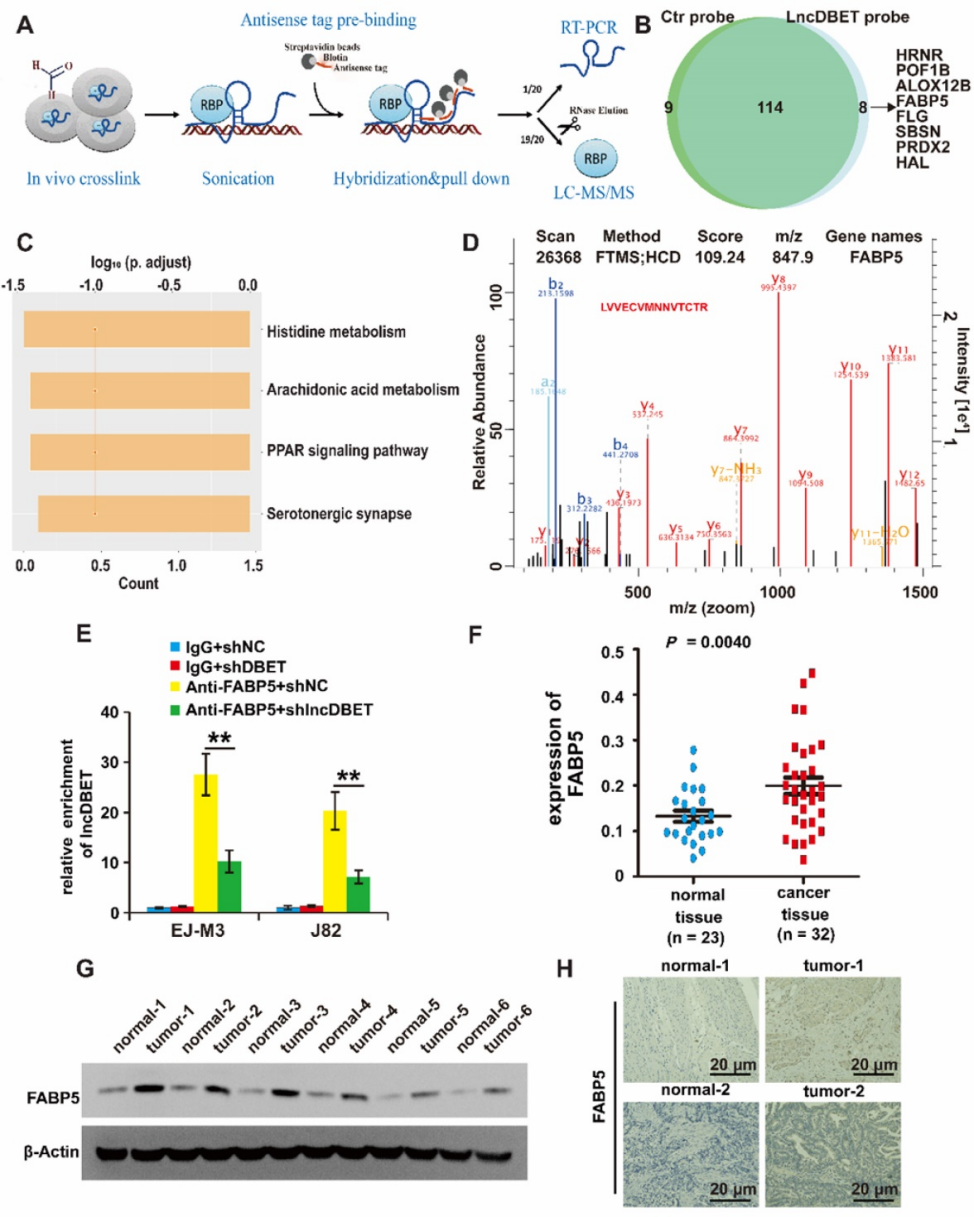
** Identification of downstream target proteins of lncDBET in BCa.** (A) The work flow diagram of ChIRP/MS used to screen the downstream target proteins of lncDBET in BCa (Referring to Yuxing Zhu's figure^25^). (B) Downstream candidates of lncDBET by ChIRP/MS (|FC| ≥ 2, P < 0.05). (C) KEGG pathway analysis of possible signaling pathways that lncDBET may be involved in. (D) MS-identified peptide sequence of FABP5. (E) FABP5-RIP-qPCR analysis of lncDBET regulated by FABP5 in EJ-M3 and J82 cell lines. (F) qPCR assay analysis of FABP5 expression in BCa tissues. (G) Western blotting analysis of FABP5 in six paired cancer and adjacent normal tissues of BCa. β-Actin was used as the control. (H) Immunohistochemistry assay analysis of FABP5 expression in two paired cancer and adjacent normal tissues of BCa. Each experiment was repeated a minimum of three times. The symbol * denotes a significant difference (P < 0.05), while ** represents a highly significant difference (P < 0.01).

Of note, lncDBET was distributed in both the cytoplasm and nucleus, suggesting that it can exert its function by various possible mechanisms, including playing pathophysiological roles by binding to proteins. Thus, we introduced a ChIRP/MS assay to systematically screen the candidate targets of lncDBET 25 (Figure 5A). Enrichment of proteins by lncDBET between lncDBET probe-treated cells and control probe-treated cells was presented, and 8 candidates (HRNR, POF1B, ALOX12B, FABP5, FLG, SBSN, PRDX2, HAL) were identified as potential lncDBET targets (|FC| ≥ 2, *P* < 0.05) (Figure 5B). Taking FC and binding score (Table S5), KEGG pathway analysis (Figure 5C) and association with human cancers into account as screening standards 26, only the PPAR signaling pathway was related to BCa, and thus, only FABP5 was selected for further verification. In addition, the peptide sequence of FABP5 “LVVECVMNNVTCTR” was identified (Figure 5D). Moreover, the results of FABP5 RIP-qPCR assay revealed that FABP5 recruited lncDBET transcripts (Figure 5E).

To determine whether FABP5 was upregulated in BCa, the expression of FABP5 in BCa samples and cell lines was detected, and the results revealed that FABP5 was upregulated in BCa (Figure 5F-G, Figure S2F-G). The FISH results demonstrated that FABP5 expression was significantly increased in BCa tissues (Figure 5H).

Collectively, these results revealed that FABP5 is a downstream target of lncDBET in BCa.

### FABP5 promotes the malignant progression of BCa

**Figure 6:**
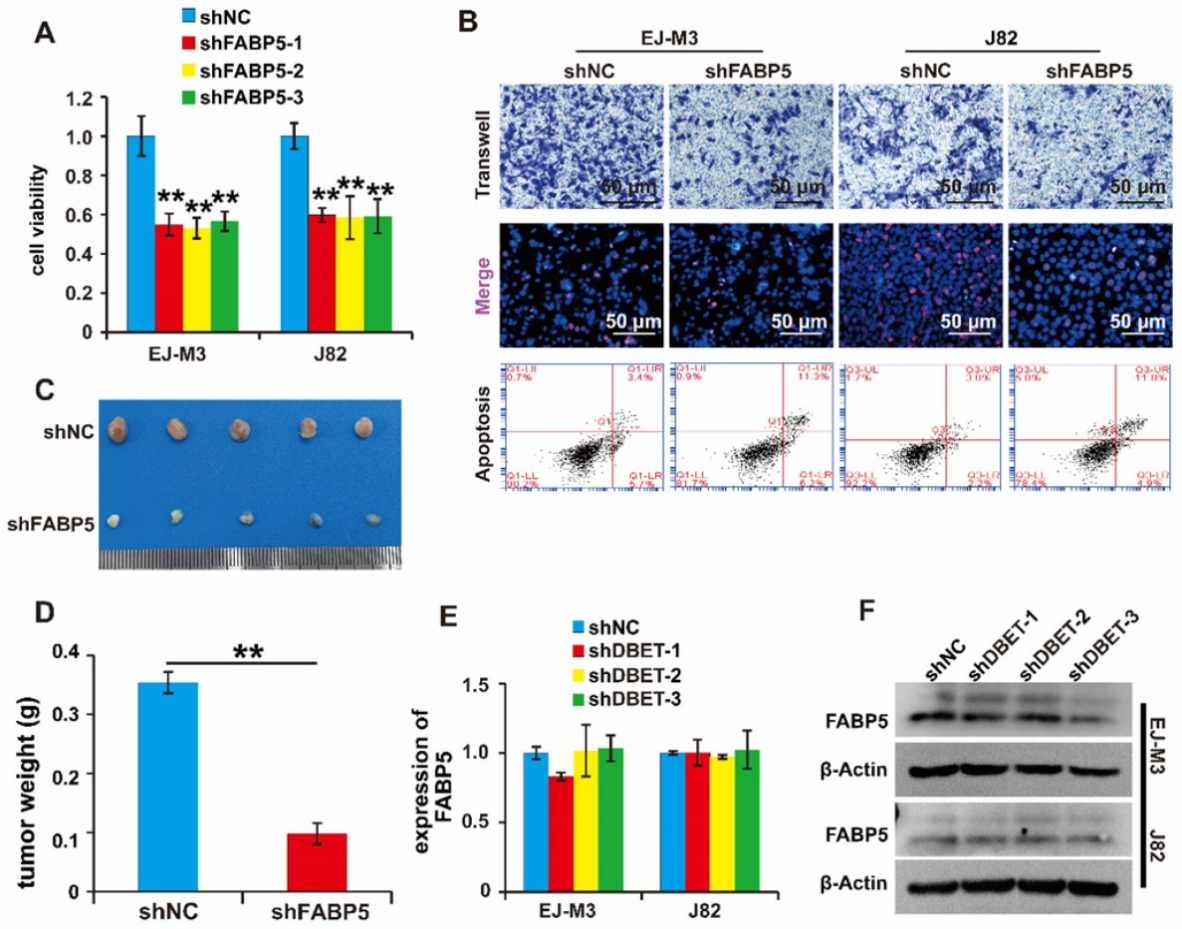
**Functional analysis of the lncDBET/FABP5 pathway in progression of BCa.** (A) CCK8 assay for the viability of EJ-M3 and J82 cell lines transfected with shFABP5. (B) Transwell, EdU and flow cytometry assays for the migration, proliferation and apoptosis of EJ-M3 and J82 cell lines transfected with shFABP5 and control shRNA (shNC). (C) Representative images of the xenograft tumors in Balbc nu/nu nude mice subcutaneously injected EJ-M3 cells transfected with shFABP5 or shNC. (D) The relative weight of tumors in Balbc nu/nu nude mice subcutaneously injected EJ-M3 cells transfected with shFABP5 or shNC. (E-F) qPCR and western blotting analysis of FABP5 regulated by lncDBET in EJ-M3 cell lines transfected with shlncDBET. Each experiment was repeated a minimum of three times. The symbol * denotes a significant difference (P < 0.05), while ** represents a highly significant difference (P < 0.01).

Of note, FABP5 directly interacted with lncDBET and was overexpressed in BCa. We speculated that FABP5 serves as a tumour promoter in BCa. To determine the function of FABP5 in BCa, we constructed a FABP5 silencing system (Figure S2H-I). Then, the effects of FABP5 expression changes on cell viability, proliferation, migration and apoptosis were measured. As expected, the depletion of FABP5 significantly inhibited BCa cell viability, proliferation and migration and promoted BCa cell apoptosis (Figure 6A-B). Furthermore, silencing FABP5 resulted in a significant decrease in tumour volume and weight (Figure 6C-D). collectively, these results indicate that FABP5 promotes the malignant progression of BCa.

Furthermore, we wondered whether lncDBET affected FABP5 expression. The qPCR analysis results demonstrated that there was no alteration of FABP5 expression in cells with lncDBET knockdown, suggesting that their expression levels are independently regulated (Figure 6E-F).

### The METTL14/lncDBET/FABP5 pathway affects the progression of BCa via the PPAR pathway

Multiple studies have shown that peroxisome proliferator-activated receptors (PPARs, including PPARα, PPARβ and PPARγ), markers of lipid metabolism-related signalling pathways, are activated by FABP5 and are involved in the progression of human cancers. To determine whether FABP5 regulates PPAR expression in BCa, the expression levels of PPARα, PPARβ and PPARγ were measured after inhibition of FABP5 expression. The results revealed that silencing FABP5 inhibited the expression of PPARs (Figure S3A-D).

**Figure 7:**
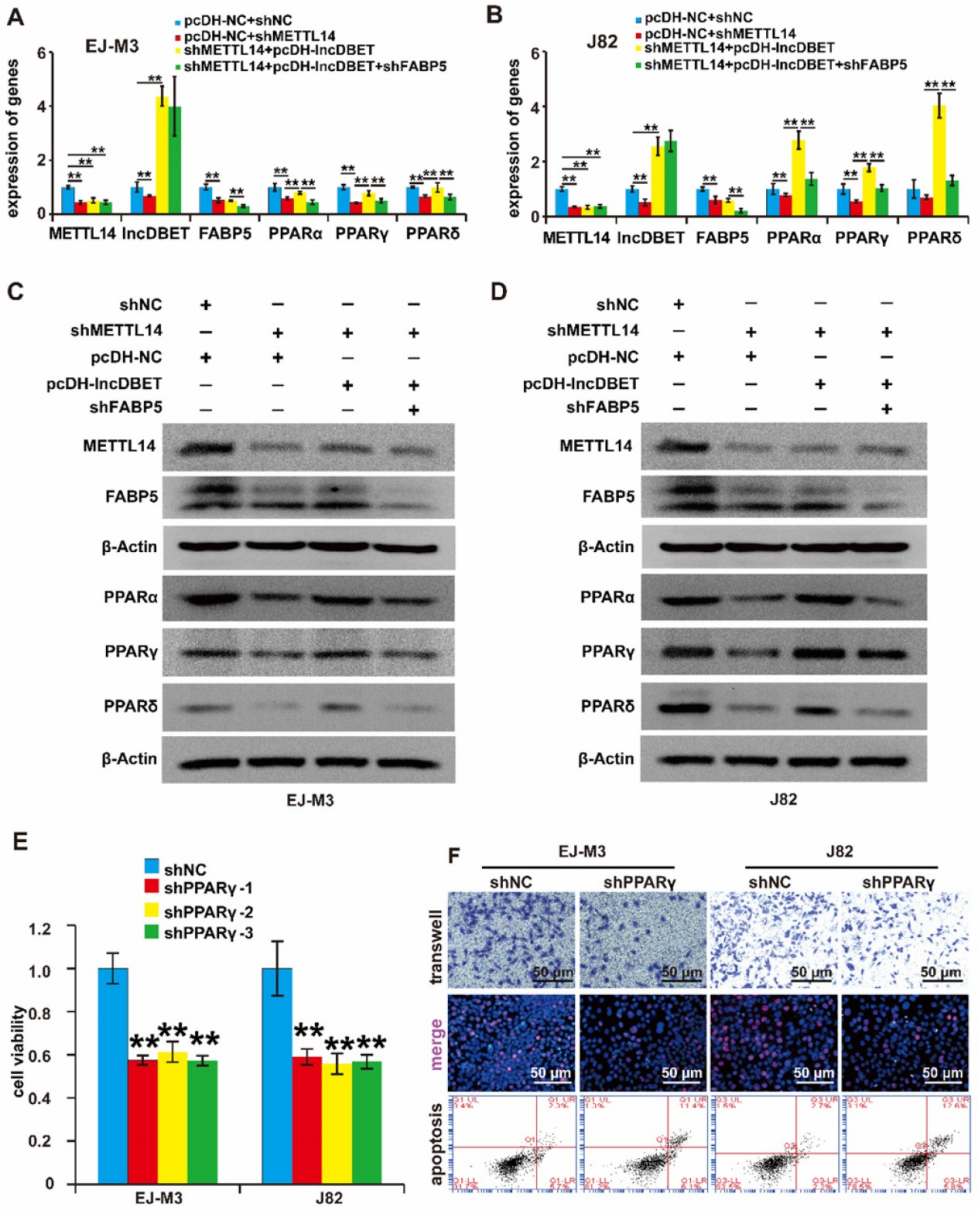
** Functional analysis of the METTL14/lncDBET/FABP5 pathway in the regulation of PPARγ.** (A-B) qPCR analysis of METTL14, DBET, FABP5 and PPARs in EJ-M3 and J82 cell lines transfected with pcDH-NC+shNC, pcDH-NC+shMETTL14, shMETTL14+pcDH-lncDBET and shMETTL14+pcDH-lncDBET+shFABP5. (C-D) Western blotting analysis of METTL14, FABP5 and PPARs in EJ-M3 and J82 cell lines transfected with pcDH-NC+shNC, pcDH-NC+shMETTL14, shMETTL14+pcDH-lncDBET and shMETTL14+pcDH-lncDBET+shFABP5. (E) CCK8 assay for the viability of EJ-M3 and J82 cell lines transfected with shPPARγ or control shRNA (shNC). (F) Transwell, EdU and flow cytometry assays for the migration, proliferation and apoptosis of EJ-M3 and J82 cell lines transfected with shPPARγ or control shRNA (shNC). Each experiment was repeated a minimum of three times. The symbol * denotes a significant difference (P < 0.05), while ** represents a highly significant difference (P < 0.01).

To verify whether the METTL14/lncDBET/FABP5 pathway regulates PPARs, we divided the cells into four groups: shFABP5+pcDH-lncDBET+shMETTL14, pcDH-lncDBET+shMETTL14, pcDH-NC+shMETTL14 and pcDH-NC+shNC. The qPCR and western blotting assay results revealed that shFABP5 inhibited the effect of lncDBET, which restored the shMETTL14-induced inhibition of PPAR expression (Figure 7A-D). In summary, these results suggest that the METTL14/lncDBET/FABP5 pathway regulates PPARs in BCa cells.

To determine the role of PPARs in BCa, the expression of PPARγ in BCa samples and cell lines was detected, which revealed that PPARγ was upregulated in BCa (Figure S3E-F). Then, we constructed PPARγ overexpression and silencing systems (Figure S3G-J). The effects of PPARγ on cell viability, proliferation, migration and apoptosis were measured. As expected, the depletion of PPARγ significantly promoted cell apoptosis and inhibited cell viability, proliferation and migration (Figure 7E-F), which indicated that the PPAR pathway is involved in the progression of BCa.

**Figure 8:**
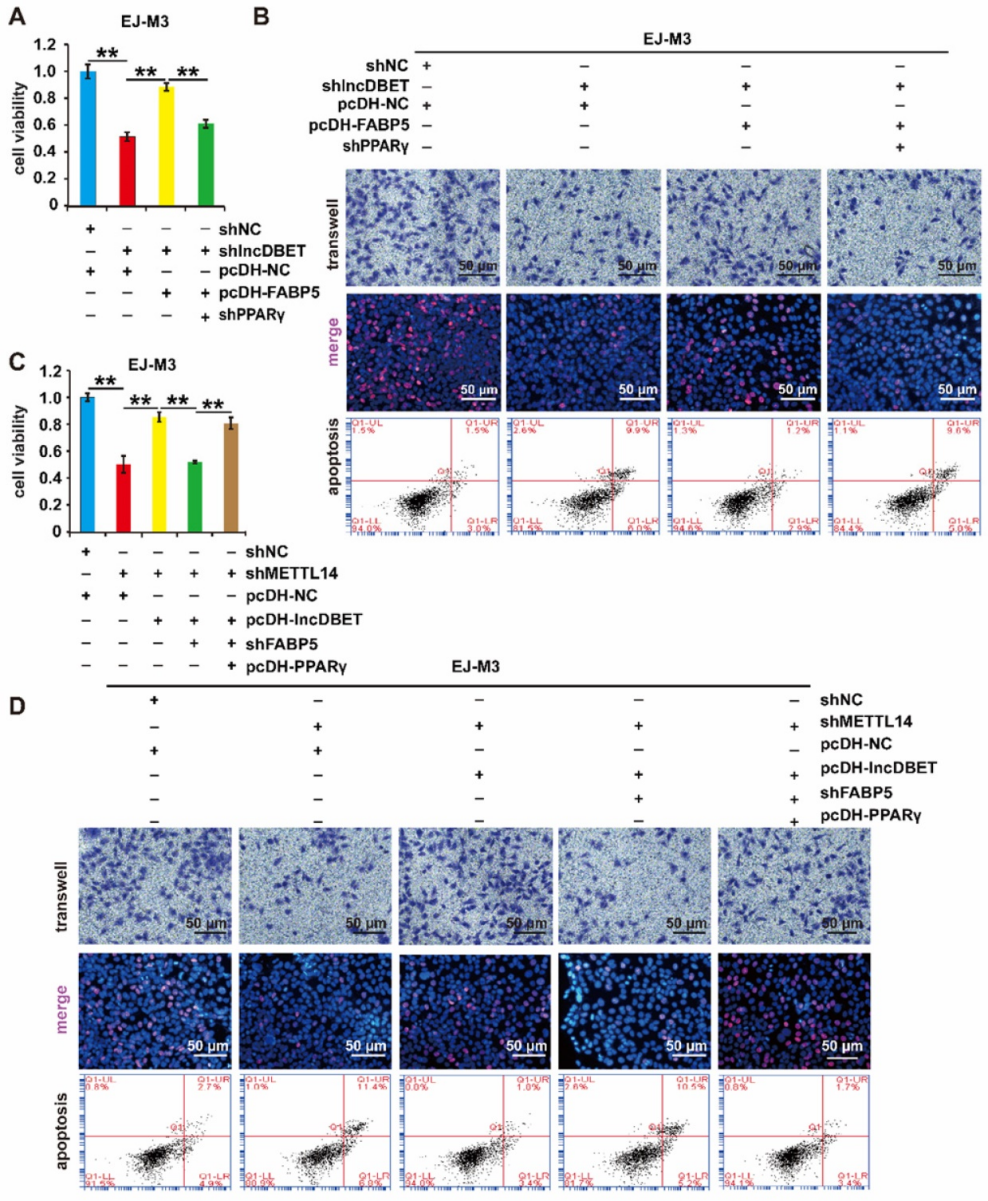
** Functional analysis of the METTL14/lncDBET/FABP5/PPARγ pathway.** (A-B) CCK8, Transwell, EdU and flow cytometry assays for the viability, migration, proliferation and apoptosis of EJ-M3 cell lines transfected with shPPARγ+pcDH-FABP5+shlncDBET, pcDH-FABP5+shlncDBET, pcDH-NC+shlncDBET and pcDH-NC+shNC. (C-D) CCK8, Transwell, EdU and flow cytometry assays for the viability, migration, proliferation and apoptosis of EJ-M3 cell lines transfected with pcDH PPARγ+shFABP5+pcDHlncDBET+shMETTL14, shFABP5+pcDH-lncDBET+shMETTL14, pcDH-lncDBET+shMETTL14, pcDH-NC+shMETTL14 and pcDH-NC+shNC. Each experiment was repeated a minimum of three times. The symbol * denotes a significant difference (*P* < 0.05), while ** represents a highly significant difference (*P* < 0.01).

The effects of PPARγ on cell viability, proliferation, migration and apoptosis were measured. As expected, the depletion of PPARγ significantly promoted cell apoptosis and inhibited cell viability, proliferation and migration (Figure 8A-B, Figure S4A-B). Furthermore, shPPARγ inhibited the effect of pcDH-FABP5 on the progression of BCa, suggesting that the lncDBET/FABP5/PPARγ pathway is involved in the progression of BCa (Figure 8A-B, Figure S4A-B).

To comprehensively analyse the function of the METTL14/lncDBET/FABP5 pathway, the cells were divided into five groups: pcDH-PPARγ+shFABP5+pcDH-lncDBET+shMETTL14, shFABP5+pcDH-lncDBET+shMETTL14, pcDH-lncDBET+shMETTL14, pcDH-NC+shMETTL14 and pcDH-NC+shNC. The results of CCK-8, EdU, Transwell and flow cytometry assays revealed that any disturbance of the pathway could affect the viability, proliferation, migration and apoptosis of BCa cells (Figure 8C-D, Figure S4C-D). Overall, these observations revealed that the METTL14/lncDBET/FABP5 pathway mediates the progression of BCa by activating the PPAR pathway.

## Discussion

The limited effect of adjuvant therapy on advanced BCa is one of the reasons for its poor prognosis. Therefore, the mechanism of the malignant progression of BCa, which is of great clinical significance for the precise treatment of BCa, needs to be further explored.

It is widely accepted that m^6^A is the most abundant posttranscriptional modification of eukaryotic mRNAs and ncRNAs 3, 27. The discovery of m^6^A methyltransferases, demethylases and binding proteins has shown that m^6^A modification is a dynamic reversible process 4. The destruction of the balance of m^6^A modification is involved in the occurrence and progression of human diseases and processes, such as cancers, obesity, reproduction, development and immune response 28. It has been reported that METTL3 and ALKBH5 can trigger the malignant progression of BCa by mediating mRNA-m^6^A modification 5, 6, 29. Although the abnormal regulation of ncRNA-m^6^A modification is also related to the progression of human cancers, its regulatory mechanism in BCa remains largely unknown 30-33. In this study, METTL14 was demonstrated to promote the malignant progression of BCa through the lncDBET/FABP5/PPAR signalling pathway. To our knowledge, this is the first study that shows METTL14-mediated promotion of the malignant progression of BCa via the regulation of lncRNA-m^6^A modification.

Our data showed that the expression of METTL14 in BCa was significantly increased and that m^6^A modification mediated by METTL14 promoted the survival, proliferation and migration of BCa cells. A previous study reported that METTL14 may be related to the progression of BCa 34. Information of the *LEE Bladder* dataset in the ONCOMINE database also proved that METTL14 expression is significantly increased in BCa. Therefore, METTL14 may participate in the tumorigenesis of BCa. METTL14, which is one of the core components of the methyltransferase complex (forming an isomeric dimer with METTL3), recognizes catalytic substrates by forming an RNA-binding scaffold. Considering that METT14 and METTL3 are isomeric dimers of each other, their expression trends should be consistent. However, several studies have reported that METTL3, instead of METTL14, is upregulated in BCa, indicating that m^6^A is a dynamic modification and that the dominant m^6^A enzyme may vary in different stages of tumour progression 35, 36.

To explore METTL14-mediated lncRNA-m^6^A modification in BCa, a microarray assay was utilized to screen candidate downstream lncRNAs. Among all the differentially expressed and differentially methylated lncRNAs, only lncDBET was proven to be upregulated based on its expression in BCa tissues and cell lines, as well as in the TCGA database. LncDBET (ENST00000630918) is the transcript of the D4Z4 binding element. It was first reported in facioscapulohumeral muscular dystrophy (FSHD), where it regulates 4q35 chromatin structure and gene derepression to cause disease 37, 38. Nevertheless, there has been inadequate research on lncDBET in human cancers.

In this study, we revealed that lncDBET upregulation promoted the malignant progression of BCa and was expressed in both the cytoplasm and nucleus. To further confirm the regulation of lncDBET by METTL14, we first assessed the level of lncDBET in MELLT14 overexpression and deletion cell models and revealed the positive regulation of lncDBET by METTL14. Second, we used MeRIP and METTL14-RIP assays to reveal that METTL14 directly interacts with lncDBET and inhibits lncDBET expression by reducing its m^6^A modification level. Third, based on the m^6^A site prediction database, luciferase reporter assays and lncRNA stability assays were used to reveal the exact mechanism by which METTL14 regulates lncDBET. METTL14 was found to increase the stability of lncDBET by modulating its m^6^A modification level to ultimately promote its expression.

LncDBET upregulation mediated by METTL14 contributes to BCa development and progression, but the details of this mechanism remain unclear. Previous reports have demonstrated that lncNRAs can play oncogenic and antioncogenic roles in human cancers by interacting with proteins 39, 40. Hence, to further investigate the transcriptional regulatory role of lncDBET in BCa, a ChIRP/MS assay was utilized to discover the interacting partners of lncDBET. Fortunately, a total of 8 candidates were identified to interact directly with lncDBET, among which FABP5 was the only candidate confirmed to play an important role in tumorigenesis. FABP5 was revealed to be upregulated and to promote malignant progression in BCa cells. Nevertheless, there was no alteration of FABP5 expression in cells with lncDBET knockdown, indicating that lncDBET does not affect the expression level of FABP5. What is the mechanism by which the lncDBET/FABP5 axis promotes the malignant progression of BCa?

Secondary programming of cell energy metabolism can promote the infinite proliferation and metastasis of cancer cells. Lipid metabolism can generally provide the necessary energy and cell metabolites for the proliferation and invasion of cancer cells, including BCa. As an important factor in lipid metabolism, FAs can provide energy for cells and play a role as signalling molecules in the metabolic regulation network. FABP5 is a member of the cytoplasmic FABP family that binds to FAs with high affinity and plays a role in the uptake, transport and metabolism of FAs. PPARs are nuclear receptor family members that regulate lipid metabolism and are also nuclear receptor transcription factors that can receive FA signals for activation 41. Previous studies have proven that the PPAR signalling pathway is active in BCa 42. In our study, PPARs were proven to be upregulated and involved in promoting the malignant progression of BCa.

Collectively, these results show that the PPAR signalling pathway can be activated by FABP5, which recruits FAs to promote the malignant progression of BCa. We speculate that lncDBET promotes the recruitment of FAs to activate the PPAR signalling pathway by affecting the posttranslational regulation of FABP5, leading to the malignant progression of BCa, but this still needs to be further verified.

In conclusion, we demonstrated that m^6^A modification mediated by METTL14 promotes the malignant progression of BCa by promoting the expression of lncDBET. Upregulated lncDBET activates the PPAR signalling pathway to promote the lipid metabolism of cancer cells through direct interaction with FABP5, thus promoting the malignant progression of BCa.

## Materials and methods

### Patients and samples

Fresh tumour tissues and corresponding adjacent nontumor specimens were collected from BCa patients who underwent radical cystectomy or electric resection at Xiangya Hospital, Central South University. The tissue samples were immediately snap-frozen in liquid nitrogen and preserved in a -80 °C freezer. Written informed consent was obtained from all the patients, and the research was supervised and approved by the Ethical Committee of Xiangya Hospital. Immunohistochemical experiments were carried out by Wuhan Sevierbio Company (Wuhan, China).

### Cell culture

Normal bladder epithelial SVHUC-1 cells and BCa cell lines (UMUC3, Ej-M3, J82, 5637 and T24) were cultured in RPMI-1640 culture medium (Gibco, USA). They were routinely preserved in our lab. All culture medium types were supplemented with 10% foetal bovine serum (FBS, Gibco, NY, USA), 100 units/ml penicillin and 100 mg/ml streptomycin (Gibco, NY, USA). All the cells were grown in a humidified atmosphere at 37 °C with 5% CO_2_.

### Gene silencing, overexpression and cell transfection

Short hairpin RNAs (shRNAs) specifically targeting METTL14, lncDBET, FABP5 and PPARs were obtained from Sango Biotech (Shanghai, China). The METTL14, lncDBET, FABP5 and PPAR overexpression constructs were based on the pcDH lentivirus vector and synthesized by Generay Biotech (Shanghai, China). The plasmids were transfected into the cells using Lipofectamine 2000 according to the manufacturer's protocol (Invitrogen, Carlsbad, USA). After transfection for approximately 72 h, the medium containing the virus was harvested and added to the cancer cells. After another 72 h, the cells were used for further research. The sequences of the shRNAs are listed in Table S6.

### RNA isolation, reverse transcription and real-time PCR

Total RNA was isolated from tissues and cells using TRIzol reagent (Invitrogen, CA, USA) and resolved in RNase-free water (Sango Biotech, Shanghai, China). Total RNA was reverse-transcribed into complementary DNA using the HiFi Script cDNA Synthesis Kit (CWBIO, Beijing, China). Real-time PCR was performed by using a SYBR Green I kit (Sango Biotech, Shanghai, China). All experiments were conducted according to the manufacturer's instructions. Each reaction was run three times. The primers used for RT-PCR are listed in Table S7.

### Western blotting

Protein was extracted by using RIPA buffer (Sango Biotech, Shanghai, China), measured using a BCA protein detection kit (Sango Biotech, Shanghai, China), separated on a 10% SDS-polyacrylamide gel (PAGE) and transferred onto polyvinylidene difluoride (PVDF) membranes (Millipore, USA). Then, the membrane was blocked in 5% milk dissolved in 1× TBST at room temperature (RT) for 1 h and incubated with primary antibodies at 4 °C overnight. On the following day, the membrane was incubated in secondary antibody at RT for 1 h, and the results were visualized using an enhanced chemiluminescence detection kit (CWBIO, Beijing, China).

### Analysis of the m^6^A level, and RNA immunoprecipitation (RIP) assay

Levels of total m6A were measured using the m6A RNA methylation detection kit (Epigentek, NY, USA) according to the manufacturer's instructions. Briefly, total RNA was isolated using RNA extraction reagent (Junxin Biotech, Suzhou, China) and resolved in RNase-free water (Sango Biotech, Shanghai, China). A total of 300 ng of total RNA was collected, and the m6A level was measured according to the manufacturer's instructions.

For the RIP experiment, an RNA-Binding Protein immunoprecipitation assay (Millipore, USA) was performed to measure the enrichment of RNA transcripts by proteins. Briefly, equal amounts of RNA (5 mg) were incubated with Protein A/G beads (BioLinked in) combined with anti-m6A antibody, anti-METTL14 antibody or IgG. The immunoprecipitated lncDBET transcripts were detected using RT‒PCR analysis.

### Cell viability assay

Cells were plated into a 96-well plate at a density of 2 × 10^4^ cells/well. Ten microlitres of Cell Counting Kit-8 (CCK-8) reagent (Takara, Dalian, China) was added to each well. After incubation in a humidified atmosphere at 37 °C with 5% CO_2_ for 2 h, the absorbance at 490 nm of the cells was tested by using a microplate reader (Thermo, USA). Each reaction was run three times.

### Cell proliferation assay

Cells were seeded into a 48-well plate at a density of 5 × 10^4^ cells/well. Two hundred microlitres of 5-ethynyl-2′-deoxyuridine (EdU, Donghuan, Shanghai, China) was added to each well. After incubation in a humidified atmosphere at 37 °C under 5% CO_2_ for 2 h, the cells were fixed with 4% paraformaldehyde (PFA), permeabilized with 0.5% Triton X-100, and stained with Apollo Staining reaction liquid and Hoechst stain. The results were photographed using a fluorescence microscope (Olympus, Tokyo, Japan). Three images were randomly obtained for each reaction.

### Cell migration assay

Cells in medium supplemented with 0.5% FBS were seeded into a Transwell chamber (3422#, Corning, USA) at a density of 5 × 104 cells/well. Then, 700 μl of medium supplemented with 10% FBS was added to the wells. After incubation in a humidified atmosphere at 37 °C under 5% CO2 for 24 h, the cells in the upper surface of the chamber were scraped with cotton swabs, and the cells in the bottom surface of the chamber were stained with 0.1% crystal violet. After air drying, the migrated cells were photographed using a microscope (Olympus, Tokyo, Japan). Three images were randomly obtained for each reaction.

### Flow cytometry assay

The cells were harvested and double stained in the dark for 15 min at room temperature with an Annexin V-FITC/PI Apoptosis Kit (Multi Sciences, Hangzhou, China). Then, BD FACS Diva software V6.1.3 (BD, NJ, USA) was used to determine cell apoptosis. All procedures were based on the manufacturer's instructions, and all experiments were performed in triplicate.

### RNA stability analysis

After different treatments, actinomycin D (Sigma, 5 μg/ml) was added to the cells and incubated for different times: 0, 0.5, 1.0, 2.0 and 3.0 h. Then, the cells were harvested, total RNA was extracted, cDNA was synthesized, and the RNA abundance was measured by RT‒PCR analysis.

### Fluorescence in situ hybridization (FISH) assay

Specific FISH probes for lncDBET were designed and synthesized (Servicebio Technology, Wuhan, China). The hybridization assay was performed in EJ-M3 cells, fresh tumour tissues and corresponding adjacent nontumor specimens. All images were analysed on a fluorescence microscope (ZKX53; Olympus).

### Nuclear-Cytosol Fractionation assay

A nuclear-cytosol RNA fractionation assay was performed using a Nuclear and Cytoplasmic Separation Kit (Junxin Biotech, Suzhou, China) according to the manufacturer's protocol. Briefly, the cells were washed with PBS buffer, and 100 µl of prechilled cytoplasmic extraction reagent I was added to every 1X106 cells to resuspend the cell pellet. The mixture was vigorously oscillated for 15 seconds at the highest speed with a shaker to completely resuspend the pellet and placed in an ice bath for 10 minutes. Prechilled cytoplasmic extraction reagent II was added and oscillated at a high speed for 5 seconds with an oscillator and then placed in an ice bath for 1 minute. The mixture was centrifuged at 16000 × g for 5 minutes, and the supernatant was the cytoplasmic extract. Cell nucleus extraction reagent was added to the precipitate, oscillated at high speed for 5 seconds and centrifuged at 16000 × g for 10 minutes. Total RNA was isolated with TRIzol reagent (Invitrogen, CA, USA).

### Luciferase reporter assay

The predicted m6A site in lncDBET was mutated (Mut). lncDBET-Mut and lncDBET-Wt were cloned into the luciferase reporter plasmid psiCHECK-2 (Promega). Then, the different luciferase reporter plasmids were transfected into the cells with or without repressed METTL14 using transfection reagent (GenePharma, Shanghai, China) according to the protocol. Seventy-two hours later, the luciferase activities of firefly and Renilla were detected using a luciferase detection kit (Promega).

### m^6^A-lncRNA epitranscriptomic microarray

Total RNA from each sample was quantified using a NanoDrop ND-1000. The sample preparation and microarray hybridization were performed based on ArrayStar's standard protocols. Briefly, total RNA was immunoprecipitated with m6A antibody. The modified RNAs were eluted from the immunoprecipitated magnetic beads as the “IP”. The unmodified RNAs were recovered from the supernatant as “Sup”. The “IP” and “Sup” RNAs were labelled with Cy5 and Cy3, respectively, as cRNAs in separate reactions using ArrayStar Super RNA Labelling Kit. The cRNAs were combined together and hybridized onto ArrayStar Human mRNA&lncRNA Epitranscriptomic Microarray (8x60K, ArrayStar). After washing the slides, the arrays were scanned in two-colour channels by an Agilent Scanner G2505C. Agilent Feature Extraction software (version 11.0.1.1) was used to analyse the acquired array images. Differentially m6A-methylated RNAs between two comparison groups were identified by filtering with the fold change and statistical significance (p value) thresholds. Hierarchical clustering was performed to show the distinguishable m6A-methylation pattern among samples.

### Chromatin Isolation by RNA Purification/Mass Spectrometry (CHIRP/MS)

In total, 2 × 107 cells were resuspended in precooled PBS buffer and crosslinked with 3% formaldehyde at room temperature on an end-to-end shaker for 30 min. Quench crosslinking with 125 mM glycine was performed for 5 min, and the solution was centrifuged at 1000 RCF for 3 min. Prebinding probes (4 for TT, 1 for NC and PC, 100 pmol/2× 107 cells) were added to streptavidin beads for 30 min. The unbound probes were removed. The beads were mixed with cell lysate and then hybridized on an end-to-end shaker at 37 °C. Next, the beads were washed 5 times with 1 ml of prewarming wash buffer for 5 min/wash. At the last wash, 1/20 of the beads were transferred for qPCR analysis. Then, 100 µL elution buffer, 20 U benzonase and eluted protein were added at 37 °C for 1 hour. The supernatant was transferred to a new low binding Eppendorf tube, and the beads were eluted again. The crosslinked sample was reversed at 95 °C, and proteins were precipitated with 0.1% SDC and 10% TCA at 4 °C for 2 h, followed by centrifugation at top speed. The pellets were washed with precooled 80% acetone 3 times.

After tryptic digestion, LC‒MS/MS was performed. Approximately 1/2 of the peptides were separated and analysed with a nano-UPLC (EASY-nLC1200) coupled to a Q-Exactive mass spectrometer (Thermo Finnigan). Separation was performed using a reversed-phase column. The mobile phases were H2O with 0.1% FA, 2% ACN (phase A) and 80% ACN, 0.1% FA (phase B). Separation of the sample was executed with a 120 min gradient at a flow rate of 300 nL/min. Data-dependent acquisition was performed in profile and positive mode with an Orbitrap analyser at a resolution of 70,000 and an m/z range of 350-1600 for MS1. For MS2, the resolution was set to 17,500 with a dynamic first mass. The automatic gain control (AGC) target for MS1 was set to 1.0 ×106 with max IT 100 ms, and 5.0 E+4 for MS2 with max IT 200 ms. The top 10 most intense ions were fragmented by HCD with a normalized collision energy (NCE) of 27% and an isolation window of 2 m/z. The dynamic exclusion time window was 20 s.

### Mouse xenografts

All animal experiments were approved by the Research Ethics Committee of XiangYa Hospital, Central South University. For the xenograft mouse model, Balbc nu/nu nude mice (six weeks old, female) were purchased from Shanghai SLAC Laboratory Animal Co., Ltd. (Shanghai, China), and the BC cell line EJ-M3 (1×107 in 0.2 ml sterile PBS) with different treatments was injected subcutaneously into Balbc nu/nu nude mice. After 1 month, the mice were sacrificed, and the volume and weight of xenografts were measured. For the mouse model of metastasis, mice were randomly divided into two groups: shNC and shMETTL14. Briefly, 1x106 EJ-M3 cells were injected into nude mice through the caudal vein. After 6 weeks, the rats were killed, and the liver and lung metastasis rates, body weight and mortality were analysed. Liver and lung samples were fixed in 4% PFA, and HE staining was carried out by Wuhan Sevierbio company (Wuhan, China).

### Statistical analysis

All the data were analysed by SPSS statistical software (v.16.0.0, Chicago, Illinois, USA). The data are expressed as the mean ± SD. All experiments were performed at least three times. Fisher's exact probability and Student's t test were used for comparisons between groups. All P values were two-sided, *P < 0.05 was considered statistically significant, and **P < 0.01 was considered highly statistically significant.

## Supplementary Material

Supplementary figures and tables.
