# Supplementary material for: m6A-induced lncDBET promotes the malignant progression of bladder cancer through FABP5-mediated lipid metabolism
Source: Theranostics. 2022 Aug 29;12(14):6291–307. doi: 10.7150/thno.71456 (PMC9475447; doi:10.7150/thno.71456)
Supplement: Supplementary file 1 — Supplementary figures and tables. [file thnov12p6291s1.pdf]

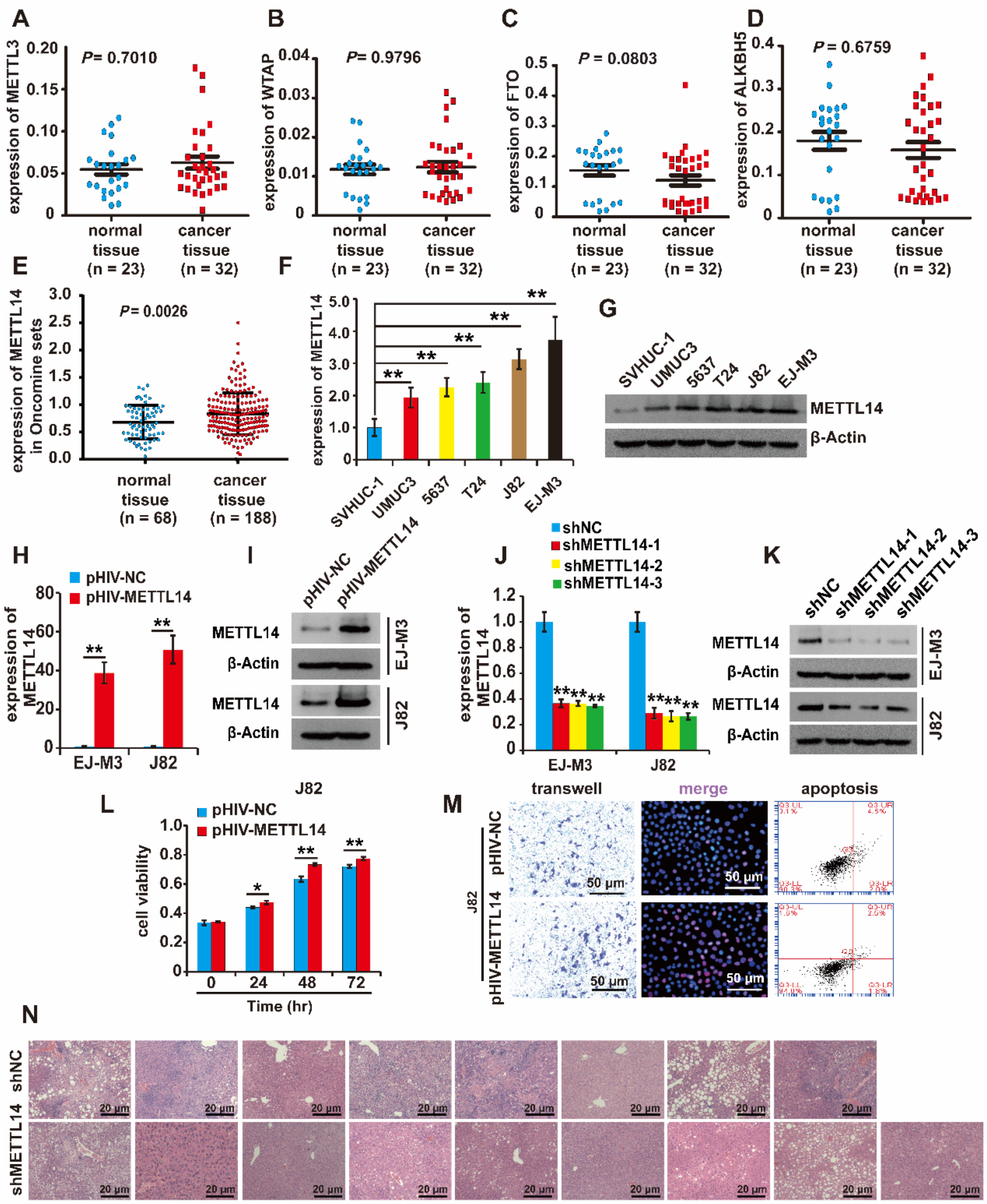

Supplementary Figure 1

(A-D) qPCR analysis of the main m^{6}A-associated methyltransferases and demethylases in BCa tissues (n = 32) and normal bladder tissues (n = 23).

(E) qPCR analysis of METTL14 in BCa tissues and normal bladder tissues from LEE Bladder in ONCOMINE database (Reporter: ILMN_1673037).

(F-G) qPCR and western blotting analysis of METTL14 in the normal urothelial cell line SVHUC-1 and five BCa cell lines (UMUC3, 5637, T24, J82 and EJ-M3).

(H-I) qPCR and western blotting analysis of METTL14 in J82 and EJ-M3 cells transfected with pHIV-METTL14 or shMETTL14.

(J-K)qPCR and western blotting analysis of FABP5 in J82 and EJ-M3 cells transfected with shMETTL14.

(L) CCK8 assay for the viability of J82 cell lines respectively transfected with shMETTL14 and pHIV-METTL14 and control shRNA(shNC)/pHIV-RNA (pHIV-NC).

(M) Transwell, EdU and Flow cytometry assays for the migration, proliferation and apoptosis of

J82 cell lines respectively transfected with shMETTL14 and pHIV-METTL14 and control shRNA (shNC)/pHIV-RNA (pHIV-NC). UL, fragment and damaged cells; UR, late apoptosis and dead cells; LL, normal cells of negative control group; LR, early apoptotic cells.

(N) HE staining analysis of the livers in metastasis tumor mice model injected EJ-M3 cells transfected with shMETTL14 or shNC through caudal vein. The bar was 20 μm. Each experiment was repeated a minimum of three times. The symbol * denotes a significant difference ($P < 0.05$), while ** represents a highly significant difference ($P < 0.01$).

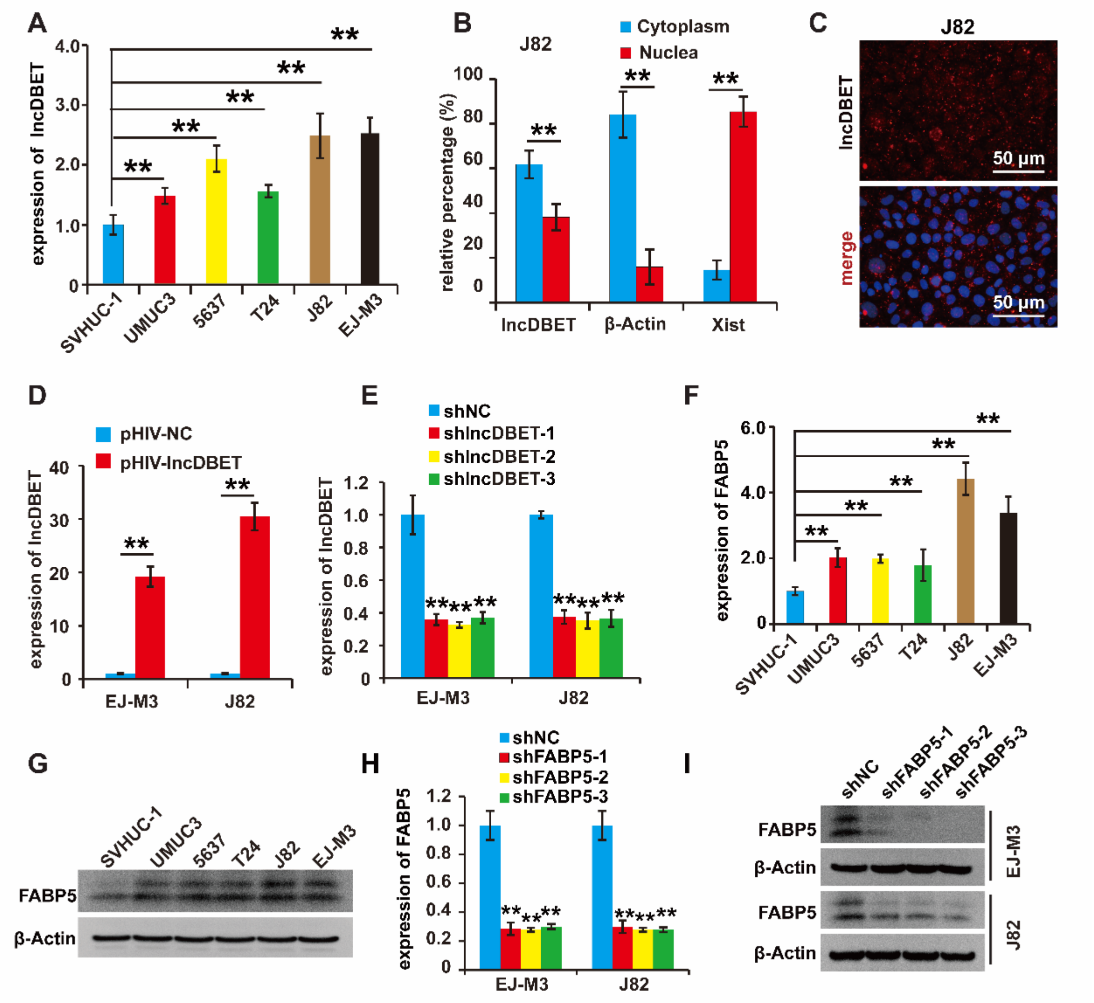

Supplementary Figure 2

(A) qPCR analysis of lncDBET in the normal urothelial cell line SVHUC-1 and five BCa cell lines (UMUC3, 5637, T24, J82 and EJ-M3).

(B) Localization analysis of lncDBET by biochemical fractionation in J82 cells. β-Actin (mostly in the cytoplasm) and Xist (mostly in the nuclea) are as control teams.

(C) Localization analysis of lncDBET by fluorescence in situ hybridization (FISH) in J82 cells. Cell nuclei were counterstained with Hoechst (blue). Each experiment was repeated a minimum of three times. The symbol * denotes a significant difference ($P < 0.05$), while ** represents a highly significant difference ($P < 0.01$).

(D-E) qPCR analysis of lncDBET in J82 and EJ-M3 cells transfected with pHIV-lncDBET or shlncDBET.

(F) qPCR analysis of FABP5 in the normal urothelial cell line SVHUC-1 and five BCa cell lines (UMUC3, 5637, T24, J82 and EJ-M3).

(G) Western blotting analysis of FABP5 in six paired cancer and adjacent normal tissues of BCa. β-Actin was used as the control.

(H-I) qPCR and western blotting analysis of FABP5 in J82 and EJ-M3 cells transfected with shFABP5.

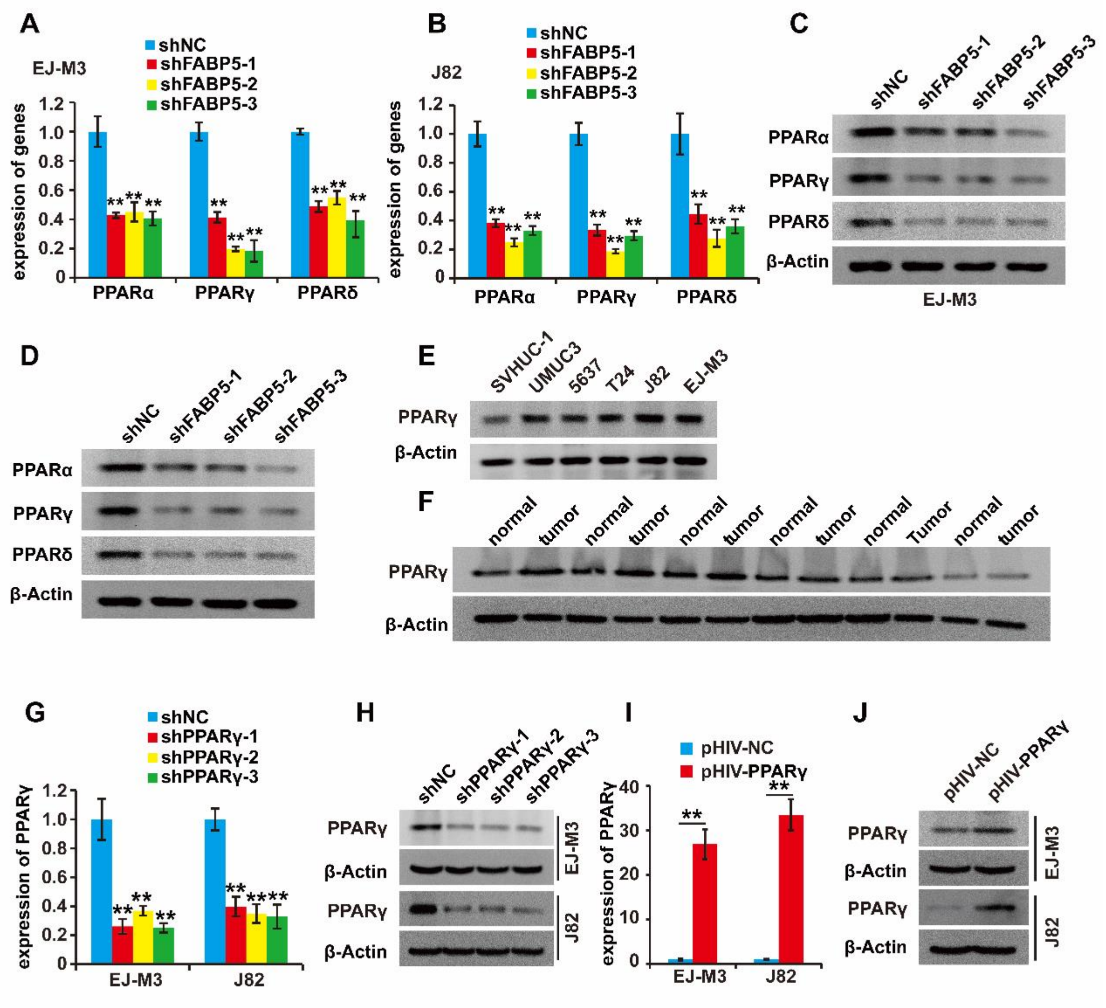

(A-D) qPCR and western blotting analysis of PPARs in J82 and EJ-M3 cells transfected with shFABP5.

(E-F) western blotting analysis of PPARγ in BCa tissues and normal bladder tissues; and in the normal urothelial cell line SVHUC-1 and five BCa cell lines (UMUC3, 5637, T24, J82 and EJ-M3).

(G-J) qPCR and western blotting analysis of PPARγ in J82 and EJ-M3 cells transfected with pHIV-PPARγ or shPPARγ.

Supplementary Figure 4

(A-B) CCK8, Transwell, EdU and flow cytometry assays for the viability, migration, proliferation and apoptosis of J82 cell lines transfected with shPPARγ+pcDH-FABP5+shlncDBET, pcDH-FABP5+shlncDBET, pcDH-NC+shlncDBET and pcDH-NC+shNC.

(C-D) CCK8, Transwell, EdU and flow cytometry assays for the viability, migration, proliferation and apoptosis of J82 cell lines transfected with pcDH PPARγ+shFABP5+pcDHlncDBET+shMETTL14, shFABP5+pcDH-lncDBET+shMETTL14, pcDH-lncDBET+shMETTL14, pcDH-NC+shMETTL14 and pcDH-NC+shNC. Each experiment was repeated a minimum of three times. The symbol * denotes a significant difference ($P < 0.05$), while ** represents a highly significant difference ($P < 0.01$).

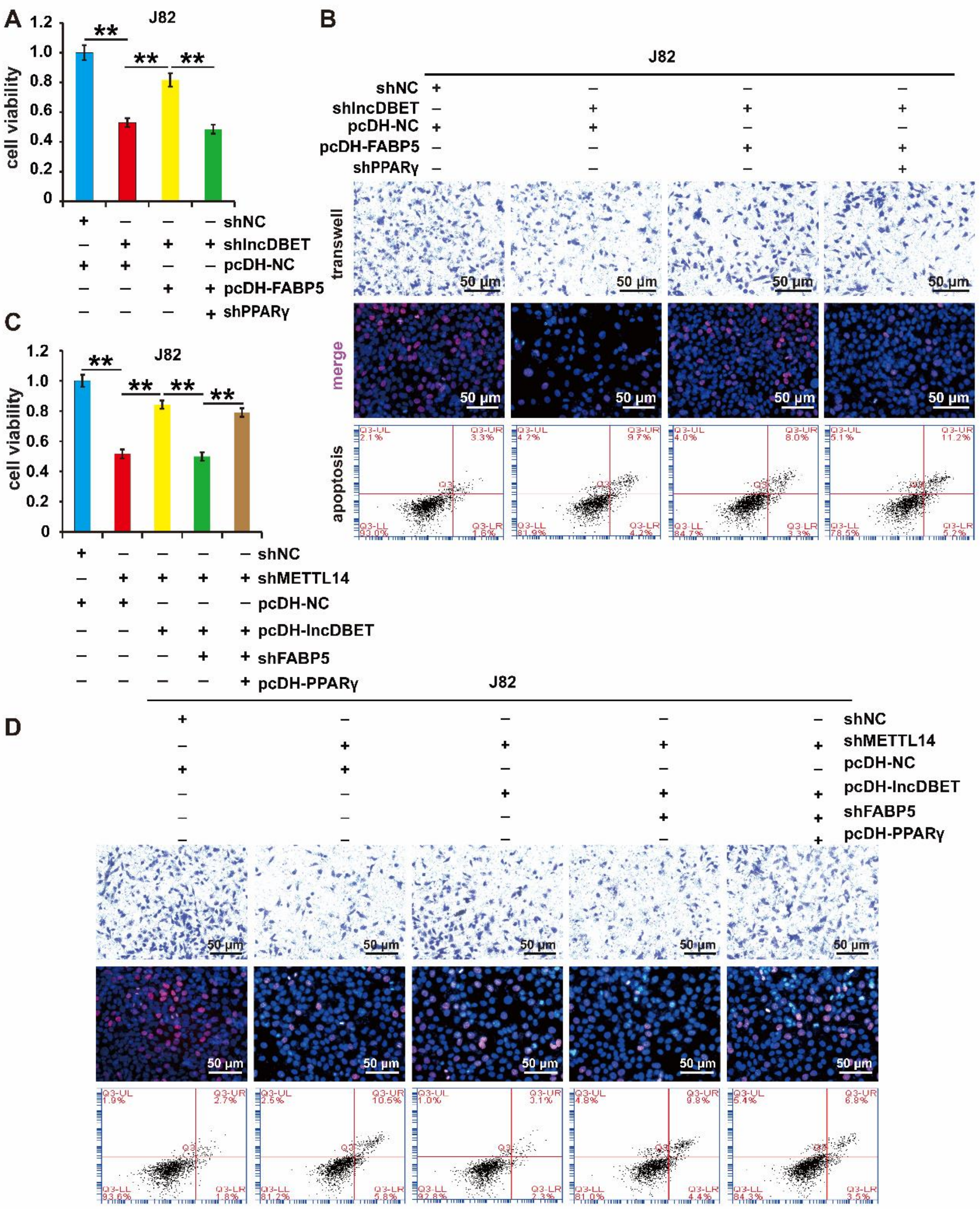

Supplementary Figure 4

(A-B) CCK8, Transwell, EdU and flow cytometry assays for the viability, migration, proliferation and apoptosis of J82 cell lines transfected with shPPARγ+pcDH-FABP5+shlncDBET, pcDH-FABP5+shlncDBET, pcDH-NC+shlncDBET and pcDH-NC+shNC.

(C-D) CCK8, Transwell, EdU and flow cytometry assays for the viability, migration, proliferation and apoptosis of J82 cell lines transfected with pcDH PPARγ+shFABP5+pcDHlncDBET+shMETTL14, shFABP5+pcDH-lncDBET+shMETTL14, pcDH-lncDBET+shMETTL14, pcDH-NC+shMETTL14 and pcDH-NC+shNC. Each experiment was repeated a minimum of three times. The symbol * denotes a significant difference ($P < 0.05$), while ** represents a highly significant difference ($P < 0.01$).

Table S1 List of downregulated m^6A modification lncRNAs from BCa m^6A-lncRNA epitranscriptomic microarray

Transcript_ID	Gene Symbol	Fold change (log2-Scaled)	P value
ENST00000508732	CTD-2536I1.1	-2.616439873	0.000113278
ENST00000561237	TRIM69	-0.850194954	0.037153064
ENST00000534036	RP11-2C23.1	-1.154659005	0.001560242
NR_045034	APH1A	-0.621146107	0.012592951
ENST00000477018	CAV2	-0.723778742	0.00886292
NR_136745	PAK3	-0.592850896	0.020527123
ENST00000599078	CTD-2561J22.4	-1.30673788	0.005164194
NR_146773	PTCSC1	-3.606802095	0.014489763
ENST00000627173	LINC00891	-1.610547232	0.019553424
ENST00000630918	DBET	-0.763805515	0.024079093
ENST00000529416	RP11-839D17.3	-0.62509301	0.049543692
ENST00000433997	RP11-92C4.3	-0.921497551	0.032014116

Table S2 List of Hypo-up lncRNAs from BCa m^6A-lncRNA & lncRNA expression microarray

Track_ID	$log_2FC.x$	$log_2FC.y$	P value	quadrant
DBET	1.4636469	-0.7613579	0.024079	Hypo-up
GS1-278J22.1	1.0886827	-1.2239234	0.169692	Hypo-up
RP11-109E24.1	1.4728784	-1.0958627	0.096076	Hypo-up
RP11-353N4.6	2.0519077	-0.8417021	0.264051	Hypo-up
RP11-84G21.1	0.9415275	-0.5917865	0.161864	Hypo-up
RP11-92C4.3	0.8763188	-0.9187676	0.032014	Hypo-up

m^6A-lncRNA epitranscriptomic microarray and lncRNA expression microarray of BCa
Hypo, down-regulated m^6A modification;
up, quantitatively over-expressed.

Table S3 Quantitative expression of the six downregulated m^{6}A modification lncRNAs from TCGA database

Gene symbol	log_2FC	P value	Significance (Y/N)
APH1A	0.558132	1.89e-04	N
LINC00891	-4.167203	1.70e-18	Y
DBET	2.821098	3.25e-04	Y
TRIM69	-0.510902	2.23e-02	N
CAV2	-0.390410	2.47e-01	N
PAK3	-0.830588	1.18e-01	N

TCGA-BLCA transcript reads count data and clinical data were downloaded using TCGA bio-links R package, and lncRNA transcripts were screened using lncRNA data provided by Gene code website. Y, yes; N, no.

Table S4 Predicted m^{6}A sites of lncDBET based on *Cui Lab*

	Position	Sequence context	Score (combined)	Decision
1	276	GUGAA AAAAU AAACU<br>GCAGU GGACA GGCUG<br>UAAGC CAGAC UUCAU	0.56	m^{6}A site<br>(**Low** confidence)
2	292	CAGUG GACAG GCUGU<br>AAGCC AGACU UCAUC<br>AAAUU UCUGC ACCAA	0.57	m^{6}A site<br>(**Moderate** confidence)
3	366	AAAAC CCUAU UAAAC<br>GUCAC GGACA AGGCC<br>AGAGU UUGAA UAUAC	0.61	m^{6}A site<br>(**High** confidence)
4	1577	UCUAG GUCCA GGCCG<br>GUGAG AGACU CCACA<br>CCGCG GAGAA CUGCC	0.54	m^{6}A site<br>(**Low** confidence)
5	1594	GAGAG ACUCC ACACC<br>GCGGA GAACU GCCAU<br>UCUUU CCUGG GCAUC	0.56	m^{6}A site<br>(**Moderate** confidence)

Table S5 Analysis of candidate proteins binding to lncDBET based on ChIRP/MS

Gene names	Score	log_2FC
HRNR	61.803	3.03212275
POF1B	28.942	2.06735177
ALOX12B	24.596	1.76272076
FABP5	21.34	1.37605871
FLG	17.028	1.37605871
SBSN	13.415	1.37605871
PRDX2	12.374	1.37605871
HAL	10.89	1.37605871

Table S6 The sequences of shRNAs

**Gene**		**Sequences**
h-METTL14-1	sense（5'-3'）	5'-CCGGGGAACTCCCAACAGGATTTCCCTCGAGGGAAATCCTGTTGGGAGTTCCTTTTTG-3'
	antisense（5'-3'）	5'-AATTCAAAAAGGAACTCCCAACAGGATTTCCCTCGAGGGAAATCCTGTTGGGAGTTCC-3'
h-shMETTL14-2	sense（5'-3'）	5'-CCGGGGATTTCCTGTGGTGGAAAGGCTCGAGCCTTTCCACCACAGGAAATCCTTTTTG-3'
	antisense（5'-3'）	5'-AATTCAAAAAGGATTTCCTGTGGTGGAAAGGCTCGAGCCTTTCCACCACAGGAAATCC-3'
h-shMETTL14-3	sense（5'-3'）	5'-CCGGGTGGTGGAAAGGAAGCTTGTTCTCGAGAACAAGCTTCCTTTCCACCACTTTTTG-3'
	antisense（5'-3'）	5'-AATTCAAAAAGTGGTGGAAAGGAAGCTTGTTCTCGAGAACAAGCTTCCTTTCCACCAC-3'
h-shDBET-1	sense（5'-3'）	5'-CCGGGCTGTCTGTGGTATTGCAGTTCTCGAGAACTGCAATACCACAGACAGCTTTTTG-3'
	antisense（5'-3'）	5'-AATTCAAAAAGCTGTCTGTGGTATTGCAGTTCTCGAGAACTGCAATACCACAGACAGC3'
h-shDBET-2	sense（5'-3'）	5'-CCGGGCAGTTTCACTAGTGCTGTAGCTCGAGCTACAGCACTAGTGAAACTGCTTTTTG-3'
	antisense（5'-3'）	5'-AATTCAAAAAGCAGTTTCACTAGTGCTGTAGCTCGAGCTACAGCACTAGTGAAACTGC-3'
h-shDBET-3	sense（5'-3'）	5'-CCGGGCACTCCACATCAGGAGAGAACTCGAGTTCTCTCCTGATGTGGAGTGCTTTTTG-3'
	antisense（5'-3'）	5'-AATTCAAAAAGCACTCCACATCAGGAGAGAACTCGAGTTCTCTCCTGATGTGGAGTGC-3'
h-shFABP5-1	sense（5'-3'）	5'-CCGGGCATGACTCTGAGGAGTTATACTCGAGTATAACTCCTCAGAGTCATGCTTTTTG-3'

	antisense（5'-3'）	5'-AATTCAAAAAGCATGACTCTGAGGAGTTATACTCGAGTATAACTCCTCAGAGTCATGC-3'
h-shFABP5-2	sense（5'-3'）	5'-CCGGGCTTCTCATCACTGAGTAAATCTCGAGATTTACTCAGTGATGAGAAGCTTTTTG-3'
	antisense（5'-3'）	5'-AATTCAAAAAGCTTCTCATCACTGAGTAAATCTCGAGATTTACTCAGTGATGAGAAGC-3'
h-shFABP5-3	sense（5'-3'）	5'-CCGGGCAGTATTCTCCCTCTCAATTCTCGAGAATTGAGAGGGAGAATACTGCTTTTTG-3'
	antisense（5'-3'）	5'-AATTCAAAAAGCAGTATTCTCCCTCTCAATTCTCGAGAATTGAGAGGGAGAATACTGC-3'
h-shPPARγ-1	sense（5'-3'）	5'-CCGGGCCCGGCTCGGCCCGACCCGGCTCGAGCCGGGTCGGGCCGAGCCGGGCTTTTTG-3'
	antisense（5'-3'）	5'-AATTCAAAAAGCCCGGCTCGGCCCGACCCGGCTCGAGCCGGGTCGGGCCGAGCCGGGC-3'
h-shPPARγ-2	sense（5'-3'）	5'-CCGGGCTCGGCCCGACCCGGCTCCGCTCGAGCGGAGCCGGGTCGGGCCGAGCTTTTTG-3'
	antisense（5'-3'）	5'-AATTCAAAAAGCTCGGCCCGACCCGGCTCCGCTCGAGCGGAGCCGGGTCGGGCCGAGC-3'
h-shPPARγ-3	sense（5'-3'）	5'-CCGGGGGTCGGCCTCGAGGACACCGCTCGAGCGGTGTCCTCGAGGCCGACCCTTTTTG-3'
	antisense（5'-3'）	5'-AATTCAAAAAGGGTCGGCCTCGAGGACACCGCTCGAGCGGTGTCCTCGAGGCCGACCC-3'

Table S7 Primers used in this study

Gene	primer	Sequence
h-METTL3	Forward	5'- TTTTCCGGTTAGCCTTCGGG -3'
	Reverse	5'- GATAGAGCTCCACGTGTCCG-3'
h-METTL14	Forward	5'- ACCTTGGAAGAGTGTGTTTACGA -3'
	Reverse	5'- TGTGAGCCAGCCTTTGTTCT-3'
h-WTAP	Forward	5'- AAATCAACTCAGTGCGGGGT-3'
	Reverse	5'- CGGGAACCCACAGTTCGATT-3'
h-FTO	Forward	5'- GTCTCCCAGGTTGATAAGGCA-3'
	Reverse	5'- GTTCGGGCAATTCGTGACTG-3'
h-ALKBH5	Forward	5'- AGTTCAGTCTTCTGCTCGCC-3'
	Reverse	5'- AGGAACTGTGGACATGGCAG -3'
h-lncDBET	Forward	5'- AAAGACCGGGACTCGGGTT -3'
	Reverse	5'- CCACGCGGAAACCAAAATCA -3'
h-FABP5	Forward	5'- TGGCCAAGCCAGATTGTATCA-3'
	Reverse	5'- CTGATGCTGAACCAATGCACC-3'
h-PPARα	Forward	5'- AAGCTGTCACCACAGTAGCTTG -3'
	Reverse	5'- AACGAATCGCGTTGTGTGAC -3'
h-PPARδ	Forward	5'- TGACAGTGTGGGGAAGTGTC -3'
	Reverse	5'- GATCCTGCATGCTCAGTCAGT -3'
h-PPARγ	Forward	5'- AGAAAACCAAGGGACCCGAA -3'
	Reverse	5'- AGAGAGGGTCCCATTTCCGA -3'
h-β-actin	Forward	5'- CATTCCAAATATGAGATGCGTTGT-3'
	Reverse	5'- TGTGGACTTGGGAGAGGACT-3'
